# Low expression of *PEBP1P2* promotes metastasis of clear cell renal cell carcinoma by post-transcriptional regulation of *PEBP1* and *KLF13* mRNA

**DOI:** 10.1186/s40164-022-00346-2

**Published:** 2022-11-08

**Authors:** Lei Yang, Haoli Yin, Yi Chen, Chun Pan, Hexing Hang, Yanwen Lu, Wenliang Ma, Xin Li, Weidong Gan, Hongqian Guo, Dongmei Li

**Affiliations:** 1 Immunology and Reproduction Biology Laboratory and State Key Laboratory of Analytical Chemistry for Life Science, Medical School, Nanjing University, Nanjing, 210093 Jiangsu China; 2 Jiangsu Key Laboratory of Molecular Medicine, Nanjing University, Nanjing, 210093 Jiangsu China; 3 Department of Urology, Affiliated Drum Tower Hospital of Medical School of Nanjing University, Nanjing, 210008 Jiangsu China

**Keywords:** *PEBP1P2*, m^5^C modification, Clear cell renal cell carcinoma, PEBP1, KLF13

## Abstract

**Background:**

Pseudogenes play an essential role in tumor occurrence and progression. However, the functions and mechanisms of pseudogenes in clear cell renal cell carcinoma (ccRCC) remain largely elusive.

**Methods:**

We quantified *PEBP1P2* expression in ccRCC tissues and cells using fluorescence in situ hybridization and real-time PCR. Besides, we evaluated the role of *PEBP1P2* in ccRCC using a lung metastasis model and a transwell assay. Finally, we documented the interactions between *PEBP1P2*, PEBP1, and KLF13 by performing luciferase, RNA immunoprecipitation, RNA pulldown, and targeted RNA demethylation assays.

**Results:**

Low *PEBP1P2* expression correlates significantly with advanced stages and poor prognosis in ccRCC patients. Besides, *PEBP1P2* overexpression inhibits ccRCC metastasis formation in vivo and in vitro. Interestingly, *PEBP1P2* directly interacted with 5-methylcytosine (m^5^C)-containing PEBP1 mRNA and recruited the YBX1/ELAVL1 complex, stabilizing PEBP1 mRNA. In addition, *PEBP1P2* increased KLF13 mRNA levels by acting as a sponge for miR-296, miR-616, and miR-3194.

**Conclusions:**

*PEBP1P2* inhibits ccRCC metastasis formation and regulates both PEBP1 and KLF13. Therefore, molecular therapies targeting *PEBP1P2* might be an effective treatment strategy against ccRCC and other cancers with low *PEBP1P2* levels.

**Supplementary Information:**

The online version contains supplementary material available at 10.1186/s40164-022-00346-2.

## Background

The most dreadful kidney tumor, renal cell carcinoma (RCC), accounts for 4% of all cancer cases worldwide [1–3]. In recent years, smoking, alcohol consumption, and obesity have increased RCC incidence. The most common form of RCC is clear cell renal cell carcinoma (ccRCC), representing 70–80% of cases [4]. In solid ccRCC tumors, the angiogenesis-related signaling pathway is activated, making them highly vascularized and setting up favorable conditions for metastasis formation [5]. Around 25–30% of patients diagnosed with cancer have metastatic or regionally advanced tumors and, in 2% of those who undergo resection, cancer recurs [6]. Because of the low sensitivity of metastatic RCC to chemotherapy and radiation, patients with metastatic RCC (mRCC) have poor outcomes. Besides, sorafenib, one of the first-line drugs against ccRCC, is less potent on mRCC [7, 8]. Therefore, it is crucial to find new therapeutic strategies against ccRCC and mRCC.

Pseudogenes accumulate evolutionary mutations [9, 10] but have no protein-coding capacity, although many are transcriptionally active [11]. The ENCODE project identified around 15,000 pseudogenes in the human genome [12]. Pseudogenes are useful to investigate the course of evolution and contain precious clues about genome dynamics [13]. Recently, a deeper understanding of the biological function of non-coding RNAs in human malignancies has emerged, and this category of natural non-coding RNAs is gathering interest [14, 15]. A growing body of literature highlights the critical roles of pseudogenes in cancers [11]. However, only a few studies based on bioinformatic analysis indicate that pseudogenes could function as essential mediators in ccRCC. These results highlight the urgency of documenting the relationship between pseudogenes and metastatic ccRCC.

Phosphatidylethanolamine binding protein 1 pseudogene 2 (*PEBP1P2*) can be transcribed into a 409 nucleotide-long non-coding RNA strand previously named lncRNA5 [16]. *PEBP1P2* inhibits abnormal proliferation, migration, and phenotypic switching in vascular smooth muscle cells during cardiovascular diseases [16]. As an important ferroptosis regulator, *PEBP1* mediates many tumor processes, including development, metastasis formation [17], and tumor microenvironment [18], for example through inflammation [19]. However, while the correlation between *PEBP1* and tumors has been proven, the role of *PEBP1P2* in ccRCC remains unclear.

Our results show that *PEBP1P2* (which is downregulated by STAT4 in ccRCC) inhibits migration, invasion, and metastasis formation. Mechanistic studies revealed that *PEBP1P2* prevents *PEBP1* mRNA decay through direct interaction and mediates *KLF13* expression via post-transcriptional regulation. Thus, this work reveals a novel regulatory mechanism involving *PEBP1P2*, a critical regulatory factor in ccRCC metastases.

## Materials and methods

### Cell culture and tissue samples

The Chinese Academy of Sciences (Shanghai, China) provided the HEK293T, HK-2, ACHN, A-498, and 786-O cells. We cultured the cells at 37 °C, under a 5% CO_2_ atmosphere, in Dulbecco’s modified Eagle medium (DMEM) with high glucose contents, 10% fetal bovine serum (FBS, Gibco, Grand Island, NY), and 1% penicillin–streptomycin (Gibco). A senior pathologist checked the patient samples collected by the Nanjing Drum Tower Hospital (Department of Pathology, Nanjing Drum Tower Hospital). Each patient provided informed consent for the use of their tissues in scientific research.

### RNA isolation and quantitative real-time PCR assays

We extracted total RNA using RNA-easy Isolation Reagent (Vazyme Biotech Co., Ltd., Nanjing, China) following the manufacturer’s instructions. Next, we reverse-transcribed RNA into complementary DNA (cDNA) and quantified it by quantitative real-time PCR (qRT-PCR) using the Vazyme HiScript II Q Select RT SuperMix for qPCR and ChamQ Universal SYBR qPCR Master Mix, respectively. We normalized quantification values using 18 s rRNA as an internal reference. We then quantified microRNA (miRNA) expression using a miRNA 1st Strand cDNA Synthesis Kit (by stem-loop) and miRNA Universal SYBR qPCR Master Mix (Vazyme). The loading control was set to U6 snRNA. Additional file 1: Table S1 lists the RNA primers.

### ChIP assay and dCas9-ChIP assay

We assessed the interaction between STAT4 and the *PEBP1P2* promoter by performing ChIP and dCas9-ChIP experiments with a ChIP Kit (Bersin Biotechnology Co., Ltd., Guangzhou, China) according to the manufacturer’s instructions. Briefly, we fixed and lysed the cells, then sonicated genomic DNA to ~ 200 bp fragments. Next, we pre-treated precipitated chromatin overnight at 4 °C with STAT4 antibody or IgG. Afterward, we purified the immunoprecipitated chromatin and analyzed it using qRT-PCR. We then transfected 786-O cells with Flag-labeled dCas9 and guide RNA targeted to the *PEBP1P2* promoter to conduct the dCas9-ChIP assay. Anti-Flag enriched the dCas9 complex containing *PEBP1P2* promoter fragments, while dCas9 enriched the proteins binding to the *PEBP1P2* promoter. Additional file 1: Table S2 lists the primers targeting potential STAT4 binding sites in *PEBP1P2* promoters.

### Dual-luciferase reporter assay

We amplified the potential STAT4-binding region of the *PEBP1P2* promoter and placed it in a pGL3-Basic vector. We also ligated the regions containing a potential miRNA response element in the 3ʹ-UTR of KLF13 mRNA to the pGLO-miR vector. Next, we transfected HEK293T cells with the indicated luciferase reporter plasmid, pRL-TK, and appropriate plasmids. Finally, we quantified the activities of the Firefly and Renilla luciferases using a Dual-Luciferase Reporter Kit (Vazyme) and normalized the Firefly luciferase activity against that of Renilla luciferase.

### RNA immunoprecipitation (RIP) and MS2-RIP assay

We performed the RIP assays using a RIP kit (Bersin) according to the manufacturer’s instructions. We lysed the cells using the RIP lysis buffer containing protease and RNase. After centrifuging the lysate, we incubated the supernatant with specific antibody-conjugated beads overnight at 4 °C. We then thoroughly washed, eluted, and purified the binding complexes. Next, we reverse-transcribed RNA into cDNA to perform qRT-PCR. We extended the *PEBP1P2* with a 12 × MS2 stem-loop for the MS2-RIP assay. We then co-transfected cells with the MS2-GFP vector and *PEBP1P2* with a MS2 stem-loop and lysed them in RIP lysis buffer. Then, we repeated the steps as in RIP assay and analyzed the protein complex by Western blot. We also evaluated the modification levels of the target mRNA using and MeRIP kit (Bersin) per the manufacturer’s instructions. Finally, we analyzed the binding complexes by qRT-PCR.

### RNA pulldown assays

We conducted the pulldown assays with biotinylated *PEBP1P2* probes (Bersin). Briefly, we produced probe-coated beads by incubating beads with the probe. After lysing and centrifuging the cells, we incubated the clarified cell lysates at 4 °C overnight with a probe-coated bead mixture. After washing and purifying the RNA complexes, we analyzed RNA by qRT-PCR and proteins by Western blot.

### Western blot

First, we lysed the cells with RIPA lysis buffer (Beyotime, Shanghai, China). After centrifugation, we mixed the separated soluble fraction with 5 × loading buffer and heated it. We then separated total protein samples by sodium dodecyl sulfate–polyacrylamide gel electrophoresis and transferred them to polyvinylidene difluoride membranes (Millipore, Darmstadt, Germany). We blocked the membranes with 5% nonfat milk for 1 h at room temperature. Then, we incubated them with the primary antibodies in 3% bovine serum albumin overnight at 4 °C. Next, we incubated the membranes with the secondary antibodies and HRP-conjugated antigens at room temperature for 1 h. Finally, we revealed the bands with the chemiluminescent ECL reagent (Vazyme) and analyzed them using ImageJ software (National Institutes of Health), using ACTB as the internal control.

### *Fluorescence *in situ* hybridization (FISH)*

Servicebio synthesized FAM-labeled PEBP1P2 probes. We performed the FISH assay using a FISH kit (Servicebio) per the manufacturer’s instructions. After DAPI staining, we captured images with a Nikon DS-U3. Additional file 1: Table S3 lists the sequences of probes.

### Immunohistochemistry

We deparaffinized, rehydrated, and incubated paraffin-embedded sections with 3% hydrogen peroxide for 3 min. Next, we permeabilized them using phosphate-buffered saline with 0.3% Triton for 15 min, then blocked them with 3% bovine serum albumin solution for 1 h. We then incubated the sections with the primary antibody overnight at 4 °C and with the secondary antibody for 1 h at room temperature. Next, we washed the sections three times with phosphate-buffered saline with Tween 20 and visualized the antibody using a DAB chromogenic kit (Servicebio, Wuhan, China) according to the manufacturer’s instruction and counterstained it with hematoxylin.

### Transwell assay

We assessed the migration and invasion of tumor cells using a transwell assay. For migration, we used uncoated polycarbonate inserts (Millipore), while we used BioCoat™ inserts (BD Biosciences) for invasion. After starving the cells overnight, we filled the upper chamber with 1–5 × 10^4^ cells suspended in DMEM without FBS and the lower chamber with 500 μL of DMEM containing 10% FBS. After crystal violet staining, we counted and analyzed the positive cells under a microscope.

### Targeted RNA demethylation system

We constructed a targeted RNA demethylation system via standard procedures such as enzyme digestion, PCR, and subcloning, as previously described [20, 21]. In brief, we fused the full-length TET1, TET2, or ALKBH5 to dCas13b and added NES to control the subcellular localization of dCas13b fusions. To target PEBP1 mRNA, we designed guide RNAs (gRNAs). Next, we co-transfected target cells with the dCas13b fusions and gRNAs.

### Plasmid construction, short hairpin RNA (shRNA), antisense oligonucleotides, lentivirus, and cell transfection

We subcloned the *PEBP1P2* sequence into a pCDH vector and fused 12 × MS2 in this plasmid to perform an MS2-RIP assay. We purchased short hairpin RNA (shRNA) and lentivirus from OBiO Technology (Shanghai, China). Following the manufacturer’s instructions, we transfected the cells with the plasmids using LipoFiter 3.0 (Hanbio, Shanghai, China). The sequences are provided in Additional file 1: Tables S4 and S5.

### Animal experiment

We maintained 6-week-old NCG (NOD/ShiltJGpt-*Prkdc*^*em26Cd52*^*Il2rg*^*em26Cd22*^/Gpt) mice under specific pathogen-free conditions. The mice received an injection of 5 × 10^4^ cells via the tail vein to establish the lung metastasis model. After two months, we counted nodules on the lung surface with the naked eye and considered them as metastases. The Animal Care and Use Committee of Nanjing University approved all the procedures (Protocol Number IACUC-D2202057).

### Statistical analysis

We analyzed continuous and categorical variables using the Mann–Whitney *U* test and the *χ*^2^ test, respectively. We compared values using Student’s *t*-test and one-way analysis of variance (ANOVA). We performed the statistical analyses with SPSS 22.0 (SPSS Inc., Chicago, IL) and plotted data with GraphPad Prism 8.0 (GraphPad Software, San Diego, CA). We considered that P < 0.05 indicated statistical significance (*P < 0.05, **P < 0.01, and ***P < 0.001). All values are expressed as the mean ± standard deviation.

## Results

### Low PEBP1P2 expression levels contribute to metastasis formation in ccRCC

**Fig. 1:**
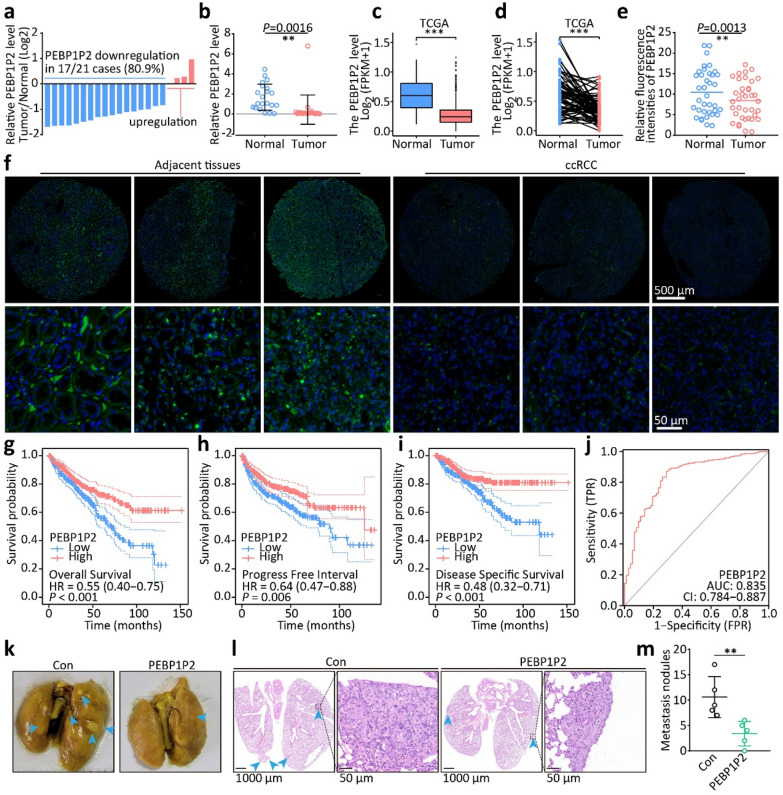
Low expression of pseudogene *PEBP1P2* contributes to metastasis of ccRCC. **a**, **b** Real-time PCR analysis of *PEBP1P2* RNA levels was conducted on 21 pairs of ccRCC tissues and corresponding non-cancerous tissues (**a**), and relative RNA level of *PEBP1P2* was normalized to internal control 18 s rRNA (**b**). **c**, **d** The RNA level of *PEBP1P2* was measured according to all samples (**c**) and the paired samples (**d**) in the ccRCC dataset TCGA database. **e**, **f** FISH detection of *PEBP1P2* (green) RNA in ccRCC tissues and its adjacent non-cancerous tissues was performed, and nuclei were visualized with DAPI counterstaining (blue). The relative fluorescence intensity was shown as a scatter plot (**e**). **g**–**i** Kaplan–Meier curve was conducted to estimate overall survival (**g**), progress-free interval (**h**) and disease-specific survival (**i**), with a 95% confidence interval (dashed lines). **j** Receiver operating characteristic (ROC) analysis was constructed for quantifying response prediction. **k** Gross view of lung samples containing metastatic nodules (cyan arrows, metastatic nodules). **l** Slides of lungs stained with hematoxylin and eosin. **m** Number of the metastatic pulmonary lesions. The data are presented as the mean ± SD, **P < 0.01, ***P < 0.001

To identify genes essential for ccRCC metastasis formation, we analyzed gene expression and prognostic data from the ccRCC dataset from The Cancer Genome Atlas (TCGA) using Gene Expression Profiling Interactive Analysis (GEPIA, http://gepia.cancer-pku.cn/) [22]. We found that a pseudogene, *PEBP1P2*, was the most important prognostic factor in ccRCC (Additional file 1: Table S1). Therefore, we evaluated the expression of *PEBP1P2* in 21 pairs of ccRCC tissues and adjacent non-cancerous tissues by qRT-PCR. We found that 17 out of 21 (80.9%) cancerous specimens had lower *PEBP1P2* RNA levels than the adjacent non-cancerous tissues (Fig. 1a and b). Analyzing *PEBP1P2* expression in the ccRCC data from TCGA and evaluating the FISH assays results on 36 paired resected specimens confirmed this finding (Fig. 1c–f).

Besides, *PEBP1P2* RNA levels were significantly correlated with advanced T stage, M stage, pathologic stage, and tumor progression (Additional file 1: Fig. S1, Table S2). Moreover, ccRCC patients with low *PEBP1P2* levels had shorter overall survival, progress-free interval, and disease-specific survival than papillary RCC and chromophobe RCC patients (Fig. 1g–i, Additional file 1: Fig. S2). Next, we determined the diagnostic utility of PEBP1P2 by performing a receiver operating characteristic (ROC) curve analysis on the ccRCC datasets. We obtained a value of 0.835 with a 95% confidence interval of 0.784–0.887 (Fig. 1j), indicating that a low *PEBP1P2* expression was correlated with poor prognosis in ccRCC patients.

To uncover the role of *PEBP1P2* deficiency in ccRCC metastasis, we silenced *PEBP1P2* in cells using shRNAs or antisense oligonucleotides, and overexpressed it using a Synergistic activation mediator (SAM) system or lentivirus vectors (Additional file 1: Fig. S3). Knocking down *PEBP1P2* markedly enhanced cell migration and invasion. Correspondingly, upregulating *PEBP1P2* notably reduced cell migration and invasion (Additional file 1: Fig. S4). In addition to the transwell assay, we established an orthotopic murine breast cancer model with an experimental tail-vein metastasis model to evaluate the role of *PEBP1P2* on metastasis formation in vivo. Five weeks after the tail vein injection, mice with tumors expressing *PEBP1P2* had significantly fewer metastatic nodules (Fig. 1k–m).

To confirm that *PEBP1P2* participated in ccRCC metastasis, we selected 786-O cells with high migratory capacity (786-O^HiMi^) and low migratory capacity (786-O^LoMi^) by performing ten rounds of transwell migration (Additional file 1: Fig. S5a). This construction method has the advantage of producing two cell lines with similar genetic backgrounds. 786-O^LoMi^ cells had higher *PEBP1P2* RNA levels than 786-O^HiMi^ cells (Additional file 1: Fig. S5b). Consistently, downregulating *PEBP1P2* in 786-O^LoMi^ cells promoted migration and invasion, and vice versa (Additional file 1: Fig. S5c). These results suggest that low *PEBP1P2* expression levels contribute to metastasis formation in ccRCC.

### STAT4 reduces PEBP1P2 expression

**Fig. 2:**
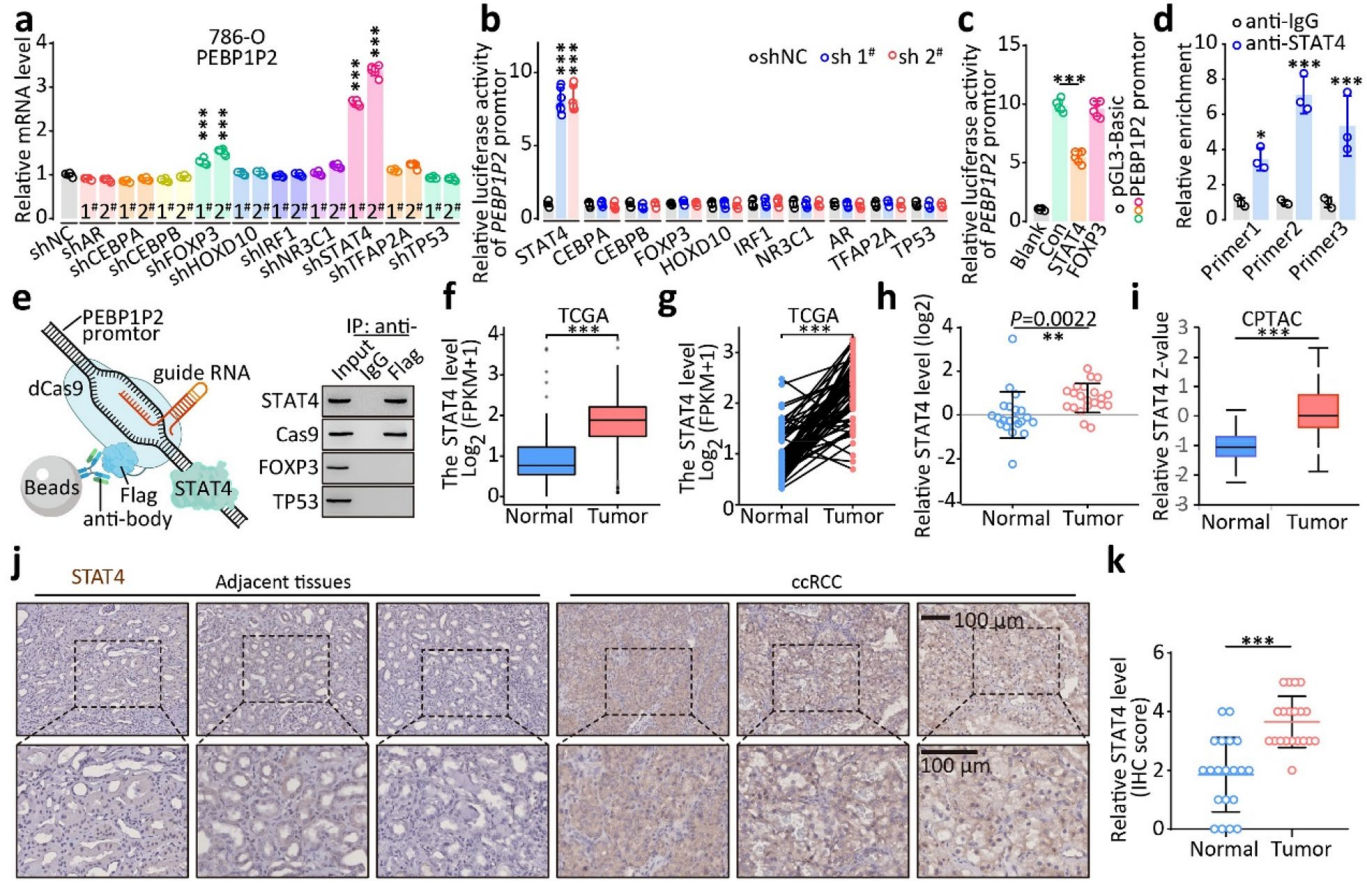
The expression of *PEBP1P2* is inhibited by STAT4. **a** Following transfection with indicated shRNAs, the RNA level of *PEBP1P2* was measured by real-time PCR. **b** Dual-luciferase reporter genes were used to evaluate *PEBP1P2* promoter region activity in HEK 293 T cells transfected with the indicated shRNAs. **c** The activity of *PEBP1P2* transcription was evaluated after transfection with indicated lentivirus via dual-luciferase reporter gene assay. **d** The binding affinity of STAT4 to *PEBP1P2* promoter in 786-O cells was determined using a ChIP assay and real-time PCR. Real-time PCR using IgG as control was also performed. **e** An overview of the dCas9-gRNA-guided ChIP (left); Western blot was performed after dCas9-gRNA-guided ChIP (right). **f**–**i** The RNA and protein level of STAT4 in ccRCC and adjacent non-cancerous tissues was analyzed according to the TCGA database (**f**,** g**), clinical ccRCC samples (**h**) and CPTAC database (**i**). **j** Representative IHC images of STAT4 in ccRCC and adjacent tissues, respectively. **k** IHC score of STAT4 in ccRCC and adjacent tissues. The data are presented as the mean ± SD, **P < 0.01, ***P < 0.001

Using the PROMO website (http://alggen.lsi.upc.es/cgi-bin/promo_v3/promo/promoinit.cgi?dirDB=TF_8.3), we analyzed the sequence of the *PEBP1P2* promoter region to investigate the upstream regulation of *PEBP1P2* [23, 24]. We thus found 21 transcription factors potentially interacting with the promoter region of *PEBP1P2*. Next, we set the differential expression as the condition (Additional file 1: Fig. S6) and screened ten genes for further investigation. Subsequently, we treated 786-O cells with lentiviruses containing target-oriented shRNA or control shRNA. The changes in *PEBP1P2* levels indicated that STAT4 and FOXP3 might be the upstream regulators of *PEBP1P2* (Fig. 2a, Additional file 1: Fig. S7a–c). Afterward, we cloned the *PEBP1P2* promoter region into a luciferase reporter plasmid and co-transfected HEK293T with it and shRNA. The dual-luciferase assays revealed that silencing STAT4 upregulated luciferase activity the most notably (Fig. 2b). Consistently, overexpressing STAT4, but not FOXP3, reduced the luciferase activity (Fig. 2c, Additional file 1: Fig. S7d). Finally, the ChIP assays confirmed that STAT4 directly bound the PEBP1P2 promoter region (Fig. 2d). Besides, the dCas9-ChIP assay revealed that STAT4, but not FOXP3, interacted directly with the *PEBP1P2* promoter region (Fig. 2e).

Based on TCGA ccRCC dataset, tumor tissues had much higher STAT4 mRNA levels than normal (Fig. 2f and g), which was further confirmed by the data from the 21 paired resected samples (Fig. 2h). Similarly, these two datasets confirmed the negative correlation between STAT4 mRNA levels and *PEBP1P2* RNA levels (Additional file 1: Fig. S7e and f). We also found that tumors expressed higher STAT4 levels than adjacent tissues in the Office of Cancer Clinical Proteomics Research (CPTAC) database and through immunohistochemistry staining (Fig. 2i–k). Moreover, silencing *PEBP1P2* rescued the inhibition of migration and invasion induced by downregulating STAT4, and overexpressing *PEBP1P2* might weaken the facilitation from the ectopic expression of STAT4 (Additional file 1: Fig. S7g). To sum up, these findings suggest that STAT4 directly represses the expression of *PEBP1P2* by binding to the *PEBP1P2* promoter region.

### PEBP1P2 stabilizes PEBP1 mRNA

**Fig. 3:**
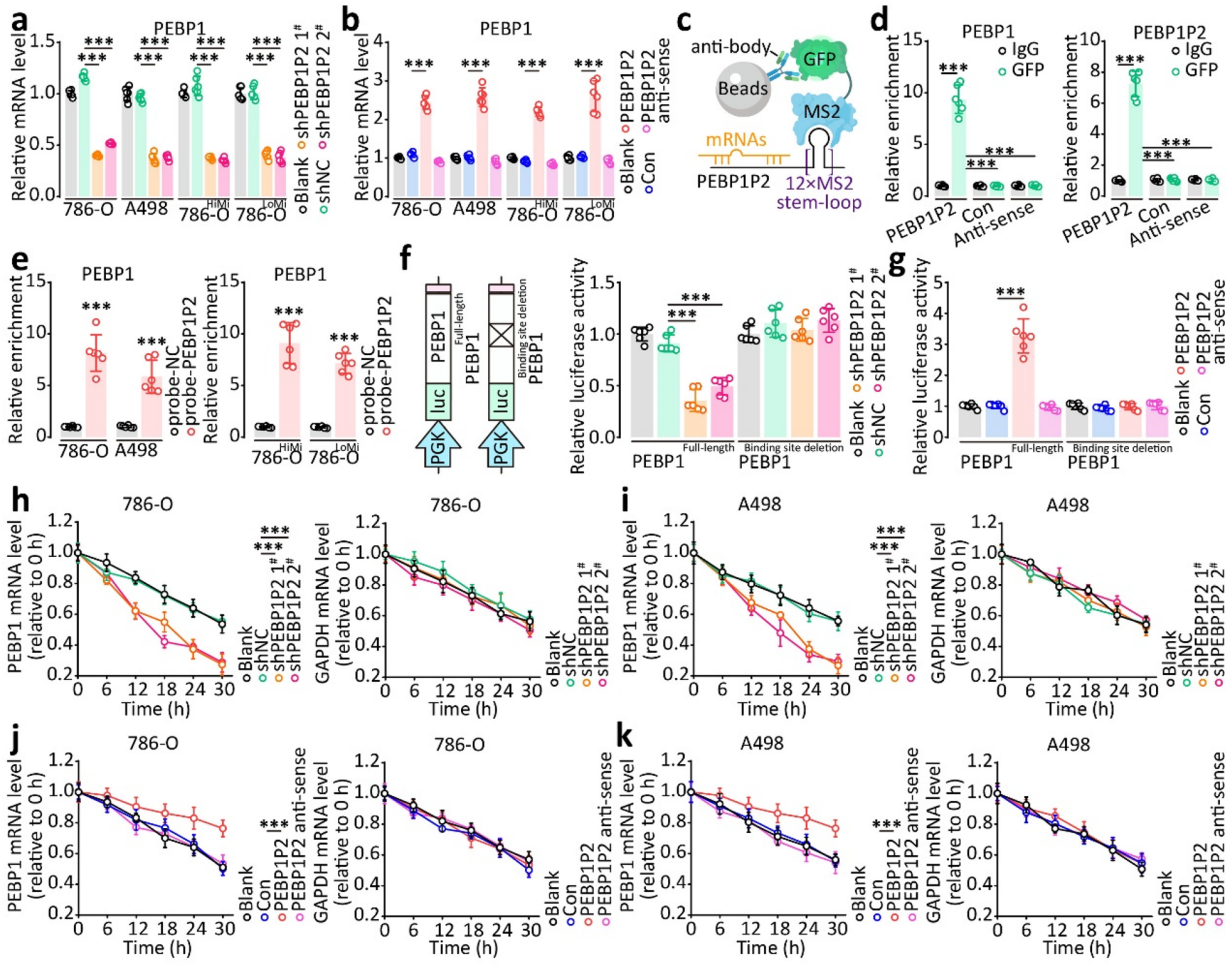
*PEBP1P2* participates in the stable maintenance of *PEBP1* mRNA. **a**, **b** *PEBP1* mRNA levels were determined after transfection with shRNAs and overexpression vector by real-time PCR. **c** Assay model for MS2-RIP. **d** Real-time PCR was performed on RNA derived from MS2-RIP samples. The result of real-time PCR was normalized based on IgG control. **e** *PEBP1* mRNA enrichment in PEBP1P2 complex was assessed by RNA pulldown assay and real-time PCR, and the mRNA enrichment was quantified by NC probe. **f**, **g** Luciferase activity of *PEBP1*-full-length and the *PEBP1*-mutation with the depletion of the binding site to *PEBP1P2* was measured after transfected with indicated lentivirus. **h**–**k** Real-time PCR was performed to determine the stability of *PEBP1* mRNA and *GAPDH* mRNA in 786-O and A-498 cells transfected with *PEBP1P2*-shRNA or overexpressing vector relative to 0 h after blocking new RNA transcription with α-amanitin. The data are presented as the mean ± SD, ***P < 0.001

To uncover the underlying mechanism of *PEBP1P2*-induced ccRCC metastasis promotion, we first analyzed the sequence of *PEBP1P2* through the Encyclopedia of RNA Interactomes (ENCORI, http://starbase.sysu.edu.cn/) [25]. In line with our conjecture, we found that *PEBP1P2* might interact with *PEBP1* mRNA (Additional file 1: Fig. S8). PEBP1 mediates ferroptosis and serves as a tumor-suppressor gene in various cancers. Knocking down *PEBP1P2* decreased PEBP1 mRNA and protein levels, and vice versa (Fig. 3a and b, Additional file 1: Figs. S9 and S10). Similarly, modulating STAT4 levels affected the mRNA and protein levels of *PEBP1* (Additional file 1: Fig. S11). Surprisingly, *PEBP1P2* did not appear to affect PEBP1 transcription or translation levels (Additional file 1: Fig. S12).

To demonstrate that *PEBP1P2* directly interacts with PEBP1 mRNA, we performed a RIP assay based on MS2 stem-loop (MS2-RIP) using the empty vector and antisense expression plasmid as a negative control. The *PEBP1* mRNA was highly enriched in the *PEBP1P2*-GFP complex (Fig. 3c and d). Besides, the RNA pulldown assay with the *PEBP1P2* probe confirmed these results (Fig. 3e). To investigate whether this direct interaction with *PEBP1P2* affected *PEBP1* mRNA expression, we cloned the full-length *PEBP1* mRNA and a *PEBP1* mRNA mutant with a deletion of the binding site of *PEBP1P2* and inserted them into the 3ʹ untranslated region (UTR) luciferase coding region (CDS). Wild-type plasmids altered luciferase activity, indicating *PEBP1P2* expression changes, whereas mutants showed no significant change (Fig. 3f and g).

Next, to explore whether *PEBP1P2* enhanced the stability of *PEBP1* mRNA, we quantified *PEBP1* and *GAPDH* in 786-O and A498 cells after treatment with α-amanitin to block RNA synthesis. Knocking down *PEBP1P2* reduced the half-life of PEBP1 mRNA, whereas overexpressing it prolonged the half-life of PEBP1 mRNA (Fig. 3h–k). The transwell assay results illustrated that overexpressing *PEBP1* reversed the inhibition of migration and invasion induced by silencing *PEBP1P2*, and vice versa (Additional file 1: Fig. S13). Aside from this, the analysis of the clinical sample and TCGA database indicated that a high *PEBP1* expression correlated with a low *PEBP1P2* expression in tumor tissues (Additional file 1: Fig. S14). These results confirm that *PEBP1P2* mediates ccRCC metastasis formation by stabilizing *PEBP1* mRNA.

### *PEBP1P2 prevents PEBP1 mRNA decay *via* RNA modification*

**Fig. 4:**
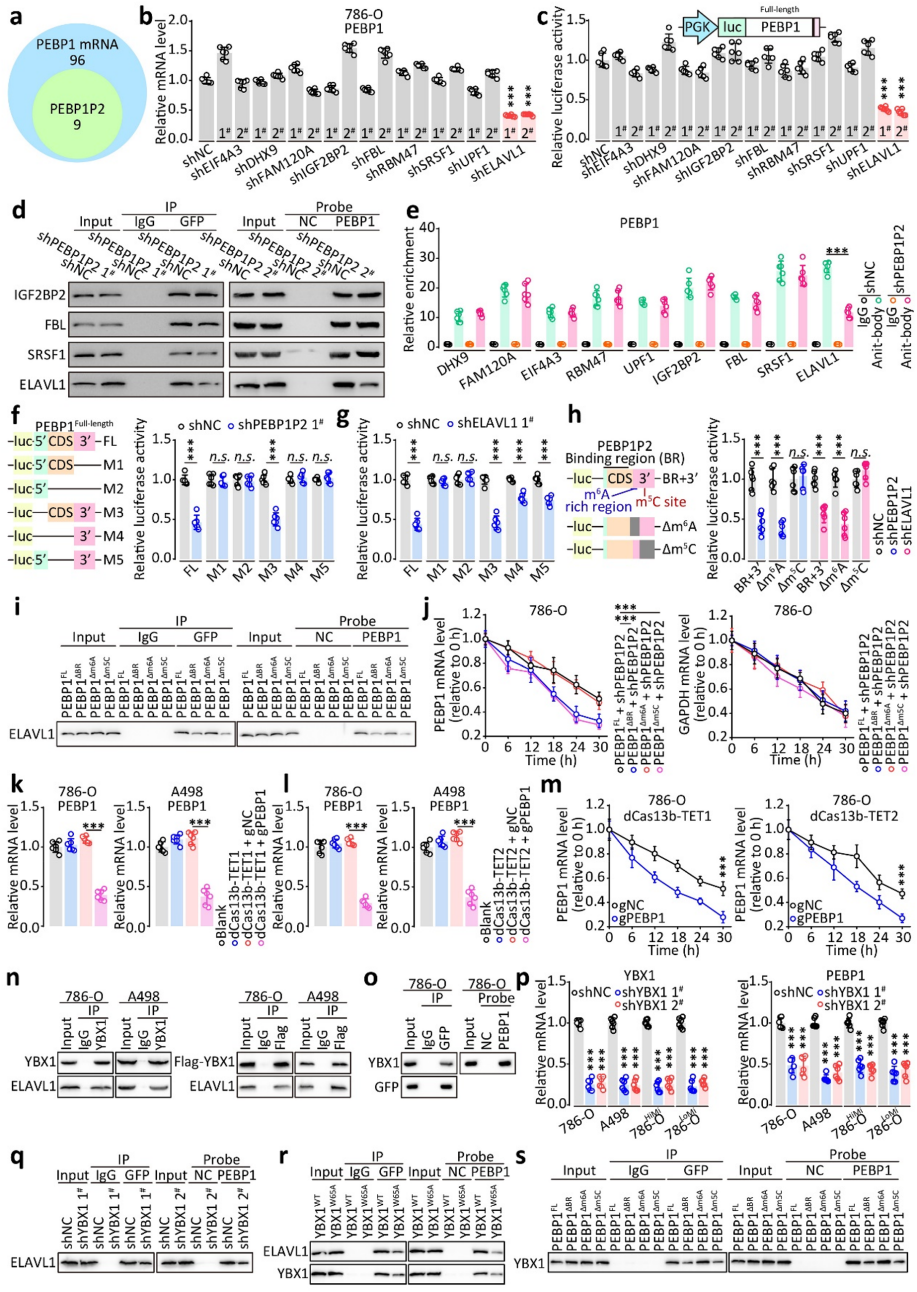
*PEBP1P2* inhibits the decay of *PEBP1* mRNA via RNA modification. **a** Venn diagram of the identified gene candidates which could interact with *PEBP1* mRNA and *PEBP1P2* predicted by ENCORI database. **b** The mRNA level of *PEBP1* after transfection with indicated shRNAs was detected by real-time PCR, respectively. **c** Luciferase activity of *PEBP1*-full-length was measured after transfected with indicated lentivirus. **d** The RNA pulldown assay, MS2-RIP, and western blot were performed to determine whether protein candidates could bind *PEBP1* RNA. **e** *PEBP1* mRNA enrichment in the complex of protein candidates was assessed by RIP assay and real-time PCR, and the mRNA enrichment was quantified by IgG control. **f**–**h** Luciferase activity of *PEBP1*-full-length and the *PEBP1*-mutation with the depletion of indicated regions was measured after being treated with indicated lentivirus. **i** The ELAVL1 binding capacity to *PEBP1* mRNA with indicated mutations was validated by MS2-RIP, RNA pulldown assay, and western blot. **j** Real-time PCR was used to determine the stability of *PEBP1* and *PEBP1* with indicated mutations and *GAPDH* transcripts in 786-O cells transfected with *PEBP1P2*-shRNA. **k**,** l** Following transfection with indicated lentiviruses, *PEBP1* mRNA levels were determined by real-time PCR. **m** Real-time PCR was used to assess the stability of *PEBP1* mRNA in 786-O cells transfected with indicated lentiviruses. **n** ELAVL1 and YBX1 were tested for interaction using the CoIP assay. **o** The interaction between YBX1 and *PEBP1* mRNA was elevated using MS2-RIP, RNA pulldown assay, and western blot. **p** The mRNA levels of *YBX1* and *PEBP1* after transfection with shYBX1 were measured by real-time PCR. **q**–**s** After transfection with indicated lentiviruses, MS2-RIP, RNA pulldown assay, and western blot was performed to determine whether YBX1 and *PEBP1* transcripts interact. The data are presented as the mean ± SD, ***P < 0.001, *n.s.* no significance

Next, to explore the molecular mechanism of *PEBP1P2*-induced *PEBP1* mRNA decay inhibition, we identified proteins potentially binding *PEBP1P2* and *PEBP1* mRNA using ENCORI. The analysis yielded nine protein candidates (Fig. 4a). Then, we designed shRNAs of these genes and transfected them into cells. Among the candidates, shELAVL1 downregulated *PEBP1* mRNA the most (Fig. 4b, Additional file 1:Fig. S15). The luciferase assay yielded results consistent with this observation (Fig. 4c). Similarly, shELAVL1 downregulated the PEBP1 protein levels (Additional file 1: Fig. S16) and ELAVL1 reduced the half-life of *PEBP1* mRNA (Additional file 1: Fig. S17). Besides, silencing *PEBP1P2* hampered the interaction between ELAVL1 and *PEBP1* mRNA, as shown by the MS2-RIP, RNA pulldown, and RIP assay results (Fig. 4d and e, Additional file 1: Fig. S18).

As an RNA-binding protein, ELAVL1 mediates RNA processing, such as maturation and degradation, via direct binding [26] to unmodified or modified RNA [27, 28]. In the *PEBP1* mRNA sequence, we found some potential *N*^6^-methyladenosie (m^6^A), 5-methylcytosine (m^5^C), and *N*^4^-acetylcytidine (ac^4^C) modification sites. Then, we confirmed the m^6^A and m^5^C modifications on *PEBP1* mRNA by m^6^A-, m^5^C-, and ac^4^C-RIP assays (Additional file 1: Fig. S19a–c). Next, to identify with which modified *PEBP1* mRNA interacts, we constructed luciferase plasmids, each containing a different truncated fragment of *PEBP1* mRNA, and co-transfected them with shPEBP1P2 or shELAVL1 into cells. *PEBP1P2* and ELAVL1 mediated luciferase activity via the CDS and 3’-UTR of *PEBP1* mRNA, which contained a large fraction of the binding site (Fig. 4f, g). Subsequently, we deleted or mutated the potential RNA modification sites and found that the m^5^C-deletion (Δm^5^C) and C-T-mutation, but not m^6^A-deletion (Δm^6^A) or A-G-mutation, eliminated shELAVL1-induced luciferase activity reduction (Fig. 4h, Additional file 1: Fig. S19d). Besides, the Δm^5^C or binding-region-deletion (ΔBR) hindered the interaction between *PEBP1* mRNA and ELAVL1 (Fig. 4i, Additional file 1: Fig. S19e), and the *PEBP1* mRNA containing Δm^5^C or ΔBR had short half-lives (Fig. 4j, Additional file 1: Fig. S19f).

To confirm that the m^5^C/m^6^A modifications were critical to producing PEBP1 mRNA, we used a targeted RNA demethylation system. The use of catalytically inactivated Cas13b (dCas13b) in conjunction with m^5^C/m^6^A erasers allowed us to add m^5^C/m^6^A modifications at sites specified through Cas13 gRNA. Full-length TET1/2- or ALKBH5-fused dCas13b with decreased gRNA targeted *PEBP1* mRNA (gPEBP1) and enriched *PEBP1* mRNA in m^5^C or m^6^A modifications (Additional file 1: Fig. S19g and h). However, only dCas13b-TET1/2 with gPEBP1 downregulated *PEBP1* mRNA and impaired the interaction between *PEBP1* mRNA and ELAVL1 (Fig. 4l, Additional file 1: Fig. S19i–l). Besides, dCas13b-TET1/2 with gPEBP1 promoted the decay of *PEBP1* mRNA (Fig. 4m, Additional file 1: Fig. S19m and n).

In general, ELAVL1 binds to m^5^C by interacting with YBX1. The co-immunoprecipitation assay confirmed the interaction between ELAVL1 and YBX1 (Fig. 4n, o), and the absence of YBX1 decreased *PEBP1* RNA and protein levels and reduced *PEBP1* mRNA half-life (Fig. 4p, Additional file 1: Figs. S20, S21). Consistently, knocking down YBX1 or using YBX1 with a loss-of-function mutation weakened the interaction between ELAVL1 and *PEBP1* mRNA (Fig. 4q and r). Aside from this, *PEBP1* mRNA containing Δm^5^C/ΔBR inhibited the binding with YBX1 (Fig. 4s), just like transfection with dCas13b-TET1/2 and gPEBP1 (Additional file 1: Fig. S22). Meanwhile, ELAVL1 and YBX1 mRNA and protein levels did not significantly change (Additional file 1: Fig. S23). Overall, *PEBP1P2* inhibited the decay of *PEBP1* mRNA via m^5^C/YBX1/ELAVL1.

### PEBP1P2 mediates KLF13 by post-transcriptional regulation

**Fig. 5:**
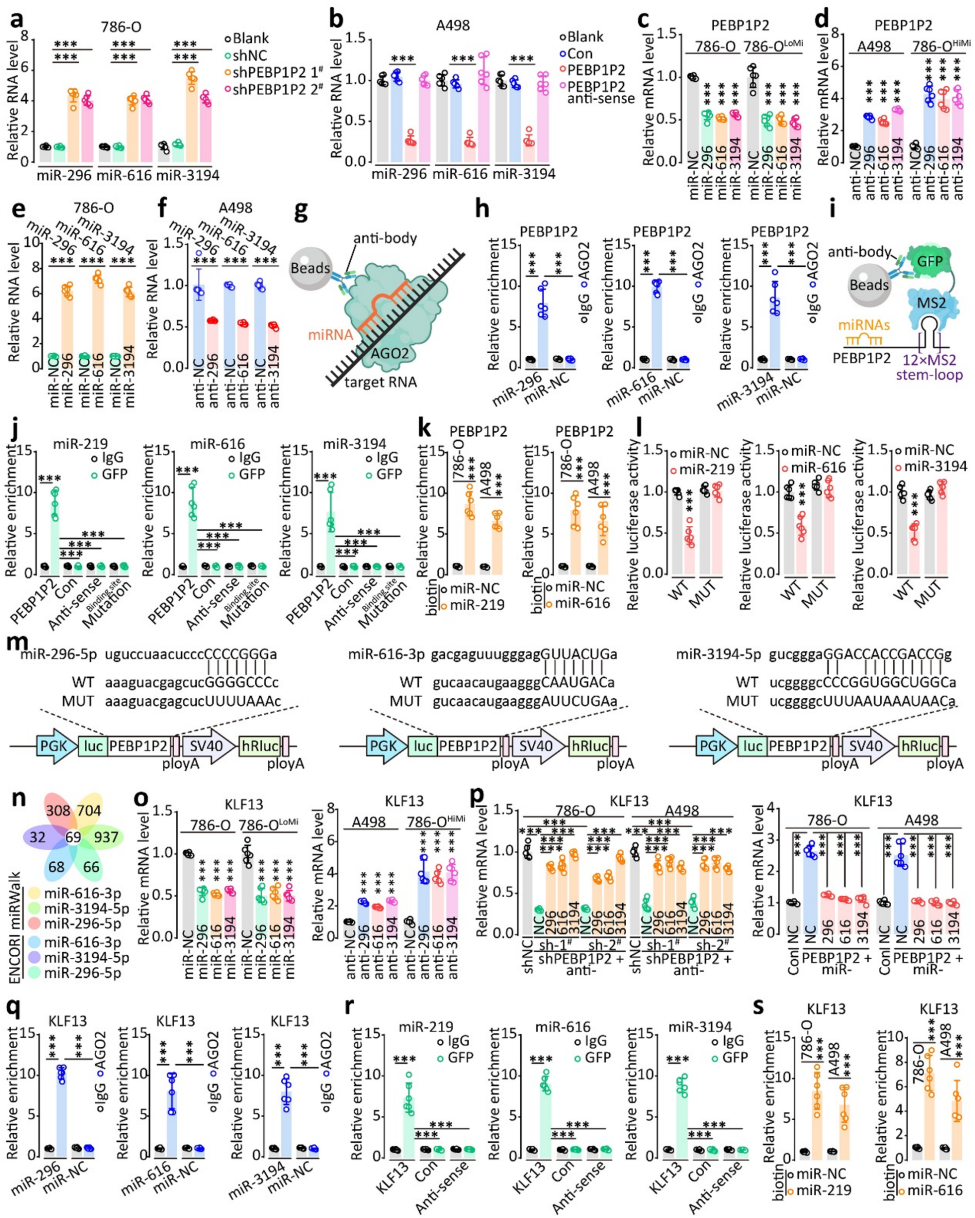
*PEBP1P2* mediates *KLF13* by post-transcriptional regulation. **a**, **b** Following transfection with indicated shRNAs or overexpressing vectors, the RNA levels of miR-296,-616, and 3194 were assessed by real-time PCR, respectively. **c**, **d** By real-time PCR, RNA levels of *PEBP1P2* were determined after transfection with mimics or inhibitors. **e**, **f** Following transfection with the indicated mimics or inhibitors, we detected the levels of miR-296/-616/-3194 by real-time PCR. **g** Assay model for AGO2-RIP. **h** AGO2 antibody was used in the RIP assay, then the enrichment of *PEBP1P2* was detected with real-time PCR. **i** Assay model for MS2-RIP. **j** RNA derived from MS2-RIP was examined by real-time PCR, with the enrichment fold-changes normalized to IgG control RNA. **k** The miRNAs-Biotin complex was enriched by Biotin-antibody with protein extracts, and the enrichment of *PEBP1P2* was measured by real-time PCR. **l-m** Transfection of HEK 293 T cells with luciferase reporter vectors and miRNA mimics was performed, and luciferase reporter activity was detected. **n** Diagram showing the selection of direct downstream targets of miR-296/-616/-3194. **o-p** By real-time PCR, the levels of mRNA of *KLF13* were determined after transfection with indicated mimics, inhibitors, and lentiviruses. **q** AGO2 antibody was used to perform RIP assays, and real-time PCR was then used to detect *KLF13* mRNA enrichment. **r** RNA derived from MS2-RIP was examined by real-time PCR, with fold changes of enrichment normalized relative to IgG control. **s** By using Biotin-antibodies in combination with protein extracts, miRNAs and biotin complexes were enriched, and the enrichment of *KLF13* mRNA was determined by real-time PCR. The data are presented as the mean ± SD, ***P < 0.001

To find out whether *PEBP1P2* could function as a miRNA sponge, we analyzed the *PEBP1P2* sequence with ENCORI and identified 14 miRNAs. After alignment with TCGA data, we found that miR-296, miR-616, and miR-3194 were upregulated in ccRCC and correlated with poor prognosis (Additional file 1: Fig. S24). Silencing *PEBP1P2* increased miR-296, miR-616, and miR-3194 levels, and overexpressing *PEBP1P2* reduced them (Fig. 5a and b, Additional file 1: Fig. S25a and b). Conversely, upregulating miR-296, miR-616, and miR-3194 decreased the *PEBP1P2* RNA levels*,* and inhibiting the miRNAs expression reduced them (Fig. 5c–f, Additional file 1: Fig. S25c, d). To confirm that their interconnected regulation depended on their direct interaction, we conducted AGO2-RIP, MS2-RIP, and RNA pulldown assays. miR-296, miR-616, and miR-3194 did directly bind to *PEBP1P2* (Fig. 5g–k, Additional file 1: Fig. S25d). A luciferase assay confirmed this conclusion (Fig. 5l and m). Besides, *PEBP1P2* appeared to play a vital role in the migration and invasion of cells by regulating miR-296, miR-616, and miR-3194 in the transwell assay (Additional file 1: Fig. S25f).

To identify the target mRNA of miR-296, miR-616, and miR-3194, we performed a conjoint ENCORI and miRWalk (http://mirwalk.umm.uni-heidelberg.de/) [29] analysis. We identified 69 mRNA candidates (Fig. 5n). Subsequently, we cloned the 3’-UTR of these potential mRNAs and inserted them into the pGLO-miR plasmid. Silencing *PEBP1P2* yielded the most notable *KLF13* activity reduction (Additional file 1: Fig. S26a). Besides, upregulating miR-296, miR-616, and miR-3194 lowered the *KLF13* mRNA levels, while transfection with anti-miR-296, anti-miR-616, and anti-miR-3194 raised them (Fig. 5o, Additional file 1: Fig. S26b and c). In addition, *PEBP1P2* regulated the *KLF13* mRNA and protein levels along with miR-296, miR-616, and miR-319 (Fig. 5p, Additional file 1: Fig. S26b–e). Next, we confirmed the direct interaction between *KLF13* mRNA and miR-296, miR-616, and miR-3194 through AGO2-RIP, MS2-RIP, and RNA pulldown assays (Fig. 5q–s, Additional file 1: Fig. S26f). The luciferase assay further confirmed this result (Additional file 1: Fig. S26g). Besides, the transwell assay results indicated that miR-296, miR-616, and miR-3194 mediated the migration and invasion of cells by regulating *KLF13* expression (Additional file 1: Fig. S27). Correspondingly, the clinical sample analysis indicated that *KLF13* expression was correlated with *PEBP1P2* expression in tumor tissues (Additional file 1: Fig. S28). Overall, these data indicate that *PEBP1P2* mediates *KLF13* expression by acting as a sponge for miR-296, miR-616, and miR-3194. According to TCGA, *PEBP1P2*, *PEBP1,* and *STAT4* were statistically associated with the prognosis of ccRCC patients, and *PEBP1P2* could serve as a diagnosis and prognosis biomarker of ccRCC (Additional file 1: Fig. S29).

## Discussion

**Fig. 6:**
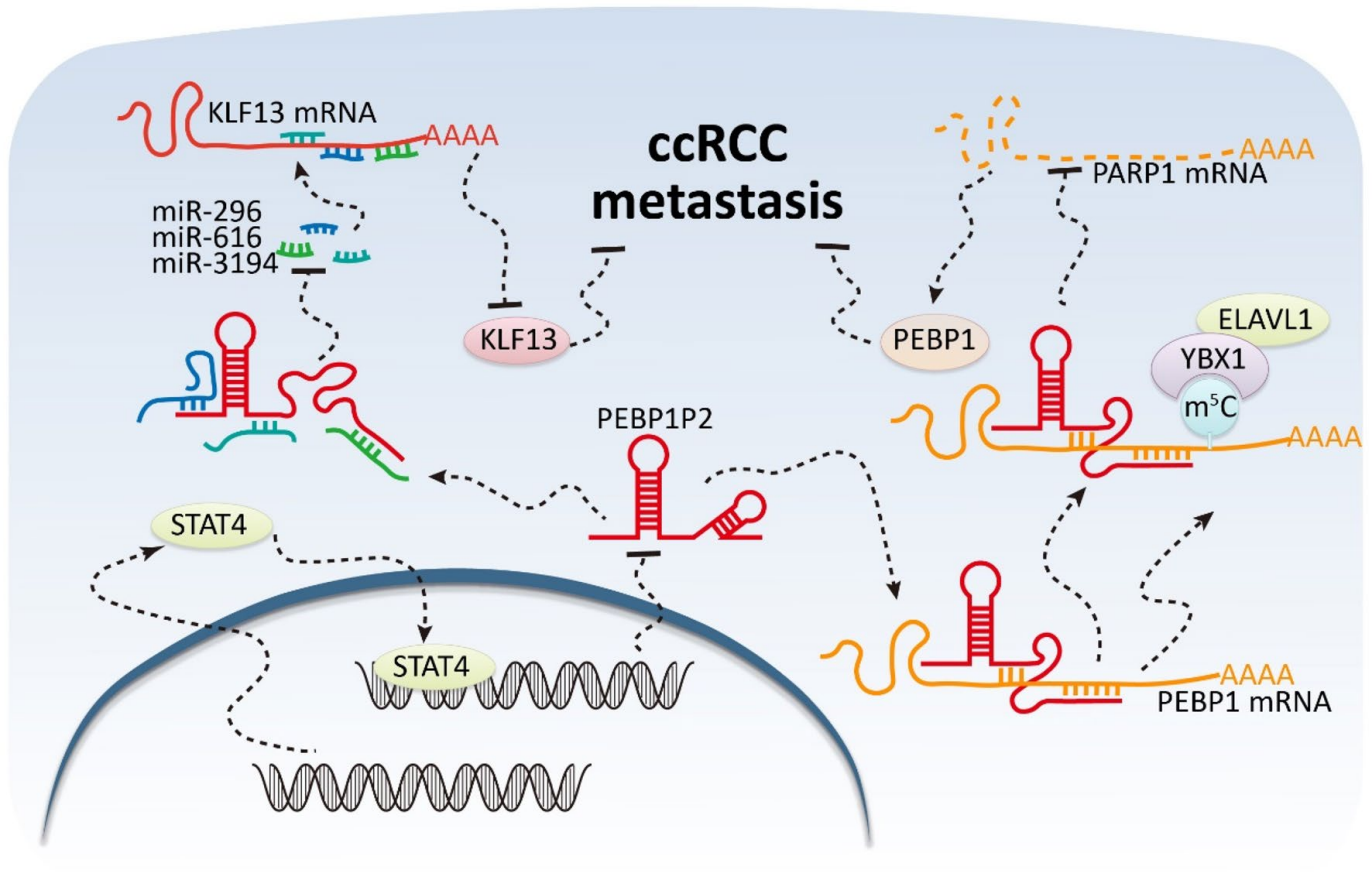
Schematic diagram for the mechanisms of *PEBP1P2* functioning as both an mRNA decay suppressor and a miRNA sponge to inhibit ccRCC metastasis

For ccRCC with rich micro-vessels and lymphatic network, metastasis formation is frequent and surgical excision is currently the primary treatment option for patients with early-stage ccRCC [5]. Unfortunately, ccRCC patients are usually diagnosed at late stages. Hence, understanding the underlying mechanisms of ccRCC metastasis formation is urgent. The present study demonstrated that, in ccRCC, the high transcription factor STAT4 levels suppress the expression of *PEBP1P2*, promoting metastasis formation. Mechanistically, *PEBP1P2* directly binds to *PEBP1* mRNA and recruits YBX1 and ELAVL1 to *PEBP1* mRNA, enhancing the stability of *PEBP1* mRNA. *PEBP1P2* also acts as a sponge for miR-296, miR-616, and miR-3194, preventing them from reducing *KLF13* expression by directly interacting with *KLF13* mRNA. Therefore, this study documents a novel ccRCC regulatory mechanism involving *PEBP1P2* and provides a new potential therapeutic strategy to overcome ccRCC metastasis formation (Fig. 6). Due to the small size of the validation cohort, our conclusions on the link between *PEBP1P2* downregulation and ccRCC patients prognosis remains limited. Thus, we aim to follow up with a prospective cohort study to detail this relationship. This future study will help develop ccRCC diagnosis and treatment tools.

Pseudogenes, once seen as leftover information from evolution, are actually involved in tumor occurrence and development [11, 14, 15]. Open reading frame disruptions lead the transcriptional products of pseudogenes, without translation products, to function like long non-coding RNA [30]. It is well known that pseudogenes contribute to forming a competing endogenous RNA network. The *UBE2CP3* pseudogene drives gastric cancer metastasis by sponging miR-138-5p and mediating ITGA2 expression [31]. Pseudogene *CTSLP8*, acting as a sponge for miR-199a-5p, promotes ovarian cancer metastasis formation [32].

Interestingly, the *WTAPP1* pseudogene promotes the translation of WTAP via direct binding with *WTAP* mRNA, facilitating pancreatic cancer progression [33]. Besides, pseudogenes can interact with proteins and regulate their expression or activation. *PRELID1P6* mediates ubiquitin-mediated degradation of hnRNPH1 and promoting glioma proliferation [34]. Similarly, *CMAHP* promotes gastric cancer metastasis formation by reducing the ubiquitination of Snail [35]. Our results show that *PEBP1P2* stabilizes *PEBP1* mRNA via direct binding and acts as a sponge for miR-296, miR-616, and miR-3194, preventing ccRCC metastasis formation.

RNA modification is an essential post-transcriptional mechanism that rapidly mediates RNA and protein levels without affecting DNA or histones. The regulation and function of the RNA methylation m^6^A in human malignancies have been studied most intensively, revealing that m^6^A affects various tumor processes [36, 37], such as proliferation [20, 21], stemness [38], and metastasis formation [39]. Besides, m^5^C mediates mRNA stability and translation, exerting powerful effects in multiple tumor types [40]. Despite the fact that NSUN family members can serve as m^5^C writers and TET1/2 participates in DNA/RNA demethylation [28, 41], very little information is available about m^5^C readers and erasers. By stabilizing the mRNA of *GRB2*, NSUN2 facilitates the development of esophageal squamous cell carcinoma [40]. The ALYREF, an m^5^C-binding protein, stabilizes *PKM2* mRNA, promoting bladder cancer tumorigenesis through PKM2-mediated glycolysis [42]. Here, we demonstrated that binding with *PEBP1P2* elongated the half-life of m^5^C-modified *PEBP1* mRNA. We revealed that the YBX1 and ELAVL1 complex stabilized *PEBP1* mRNA, probably after recruitment by *PEBP1P2*.

Most RNA modification studies knock down or overexpress proteins of interest to assess the function of m^6^A/m^5^C. However, the global effect on all potential modification sites might affect the results. Li [43] and Wilson [44] designed and established a targeted m^6^A RNA methylation system allowing to assess the effect of m^6^A modifications at specific sites without affecting the levels of the protein of interest [20, 21]. Correspondingly, we designed and used a targeted m^5^C RNA demethylation system to illustrate the effect of m^5^C at specific *PEBP1* mRNA sites.

The RNA-binding protein ELAVL1 has multiple functions via multiple binding modes. ELAVL1 directly binds to mRNA and affects its stability or alternative splicing via AU-rich elements [45, 46]. ELAVL1 could potentially function as a reader of m^6^A to mediate the stability of RNA. ELAVL1 stabilizes *ZMYM1* mRNA in an m^6^A-dependent manner, enhancing gastric cancer progression by facilitating the transition from epithelial to mesenchymal tissue [47]. METTL3 stabilizes *ARHGDIA* mRNA by modulating ELAVL1 expression in prostate cancer [27]. Here, we confirmed that ELAVL1 binds to RNA through m^5^C, and YBX1 serves as a bridge. YBX1 binds to *HDGF* mRNA in an m^5^C-depend manner, and ELAVL1 mediates the half-life of *HDGF* mRNA by interacting with YBX1 [28], promoting bladder cancer pathogenesis. Here, we showed that ELAVL1 and YBX1 increased the half-life of *PEBP1* mRNA, inhibiting ccRCC progression.

## Conclusions

In conclusion, our results demonstrate that the pseudogene *PEBP1P2* significantly impacts ccRCC metastasis formation. A high STAT4 expression lowers *PEBP1P2* expression, preventing *PEBP1P2* from protecting PEBP1 mRNA by binding to it directly, recruiting the YBX1/ELAVL1 complex, suppressing the expression of *KLF13* through sponging miR-296, miR-616, and miR-3194. These findings improve the understanding of the biological function and underlying mechanism of pseudogene *PEBP1P2* in ccRCC metastasis formation and provide insights for RNA-based diagnosis and therapy of advanced ccRCC.

## Supplementary Information

**Additional file 1: Fig. S1.** Low expression of pseudogene PEBP1P2 is linked to the advanced stage of ccRCC. a-d The RNA level of PEBP1P2 was analyzed according to different group in the ccRCC dataset TCGA database. The data are presented as the mean ± SD, *P< 0.05, **P< 0.01, ***P< 0.001. **Fig. S2.** Low expression of pseudogene PEBP1P2 shows no effect on the progression of papillary RCC and chromophobe RCC. a-c Kaplan–Meier curve was conducted to estimate overall survival (a), disease specific survival (b) and progress free interval (c) in papillary RCC dataset of TCGA database. d-f Kaplan–Meier curve was conducted to estimate overall survival (d), disease specific survival (e) and progress free interval (f) in chromophobe RCC dataset of TCGA database. **Fig. S3.** The RNA level of PEBP1P2 after transfection with indicated lentivirus or ASOs. a The RNA level of PEBP1P2 after transfection with indicated shRNAs. b The RNA level of PEBP1P2 after transfection with indicated overexpressing vector. c The RNA level of PEBP1P2 after transfection with indicated lentivirus or ASOs. d The RNA level of PEBP1P2 after transfection with indicated lentivirus. The data are presented as the mean ± SD, ***P< 0.001. **Fig. S4.** PEBP1P2 reduces cell migration and invasion. a-c Migration and invasion assays were conducted with transfected cells using Transwell inserts. **Fig. S5.** Low expression of PEBP1P2 participates the formation of high migratory capacity. a Ten rounds of Transwell selection were conducted to screened out the 786-O cell with high migratory capacity (786-OHiMi) and low migratory capacity (786-OLoMi), and migration and invasion assays were conducted to confirm the construction of these two cell lines. b The RNA level of PEBP1P2 after ten rounds of Transwell selection was detected by real-time PCR. c Migration and invasion assays were conducted with transfected cells using Transwell inserts. The data are presented as the mean ± SD, ***P< 0.001. **Fig. S6.** The mRNA levels of 21 transcription factors were analyzed. The mRNA level of 21 transcription factors which could bind to the promoter region of PEBP1P2 were analyzed according to the all samples and the paired samples in the ccRCC dataset TCGA database. The data are presented as the mean ± SD, *P< 0.05, **P< 0.01, ***P< 0.001, n.s. = no significance. **Fig. S7.** The expression of PEBP1P2 is inhibited by STAT4. a The mRNA level of indicated genes after transfection with indicated shRNAs was detected by real-time PCR respectively. b The RNA level of PEBP1P2 after transfection with indicated shRNAs was detected by real-time PCR. c The mRNA level of indicated genes after transfection with indicated shRNAs was detected by real-time PCR respectively. d The protein levels of STAT4 and FOXP3 were determined by western blot after overexpression of STAT4 and FOXP3. e, f The correlation between STAT4 and PEBP1P2 was analyzed according to the ccRCC dataset in TCGA database (e) and the clinical ccRCC sample (f). g Migration and invasion assays were conducted with transfected cells using Transwell inserts. The data are presented as the mean ± SD, ***P< 0.001. **Fig. S8.** The expression of potential mRNA binding with PEBP1P2 in ccRCC. a Heat map comparing the mRNA level of potential mRNA binding with PEBP1P2 in ccRCC and adjacent non-cancerous tissues. b-i Analysis of potential gene in ccRCC and adjacent non-cancerous tissues were performed using TCGA data and CPTAC data. The data are presented as the mean ± SD, *P< 0.05, **P< 0.01, ***P< 0.001. **Fig. S9.** The mRNA level of PEBP1 after transfection with indicated lentivirus or ASOs. a The mRNA level of PEBP1 after transfection with indicated ASOs. b The mRNA level of PEBP1 after transfection with indicated lentivirus. The data are presented as the mean ± SD, ***P< 0.001. **Fig. S10.** The protein level of PEBP1 after transfection with indicated lentivirus or ASOs. a The mRNA level of PEBP1 after transfection with indicated shRNAs or overexpressing vector. b The mRNA level of PEBP1 after transfection with indicated ASOs or lentivirus. The data are presented as the mean ± SD, ***P< 0.001. **Fig. S11.** The protein level of PEBP1 after transfection with indicated lentivirus. The protein level of PEBP1 after transfection with indicated lentivirus. The data are presented as the mean ± SD, *P< 0.05, **P< 0.01, ***P< 0.001. **Fig. S12.** PEBP1P2 shows no influence on the transcription and translation of PEBP1. a The protein level of PEBP1 in cells transfected with indicated shRNAs was detected after treatment of 5 μM cycloheximide (CHX) and removing CHX for indicated time periods. b The luciferase activity of PEBP1 promoter region was determined via dual luciferase assay kit after transfection with indicated shRNAs. The data are presented as the mean ± SD. **Fig. S13.** PEBP1P2 reduces cell migration and invasion via regulating the expression of PEBP1. Migration and invasion assays were conducted with cells, transfected indicated lentivirus, via Transwell inserts. **Fig. S14.** PEBP1 is low expressed in ccRCC. a The mRNA level of PEBP1 in 21 pairs of human clinical ccRCC tissues normalized to corresponding adjacent non-cancerous tissues was detected by real-time PCR. b The correlation between PEBP1P2 and PEBP1 was analyzed according to the clinical ccRCC sample. c, d The mRNA level of PEBP1 was analyzed according to the all samples (c) and the paired samples (d) in the ccRCC dataset TCGA database. e IHC score of PEBP1 in ccRCC and adjacent non-cancerous tissues. f Representative IHC images of PEBP1 in ccRCC and adjacent non-cancerous tissues respectively. The data are presented as the mean ± SD, ***P< 0.001. **Fig. S15.** Silence ELAVL1 inhibits the mRNA level of PEBP1. a The mRNA level of indicated RNA binding proteins after transfection with indicated shRNAs. b The mRNA levels of ELAVL1 and PEBP1 after transfection with indicated shRNAs. The data are presented as the mean ± SD, ***P< 0.001. **Fig. S16.** Silence ELAVL1 inhibits the protein level of PEBP1. The protein levels of ELAVL1 and PEBP1 after transfection with indicated shRNAs. The data are presented as the mean ± SD, **P< 0.01. **Fig. S17.** Silence ELAVL1 promotes the mRNA decay of PEBP1. The stability of PEBP1 mRNA and GAPDH mRNA in 786-O and A-498 cells transfected with ELAVL1-shRNA was measured by real-time PCR relative to 0 h after blocking new RNA synthesis with α-amanitin. The data are presented as the mean ± SD, ***P< 0.001. **Fig. S18.** Silence PEBP1P2 shows no effect on the interaction between PEBP1 mRNA and indicated RNA binding proteins. MS2-RIP, RNA pulldown assay and western blot were conducted to elevate the binding capacity of protein candidates to PEBP1 mRNA. **Fig. S19.** PEBP1P2 participates the stable maintenance of PEBP1 mRNA via m5C. a-c RNA derived from RIP assay with ac4C (a), m5C (b) and m6A (c) antibody was examined by real-time PCR. The levels of the real-time PCR products were normalized relative to IgG control. d Luciferase activity of PEBP1-wild-type and the PEBP1-mutation was measured after transfected with indicated lentivirus. e RNA derived from RIP assay with ELAVL1 antibody was examined by real-time PCR. The levels of the real-time PCR products were normalized relative to IgG control. g, h Schematic illustration of targeted RNA demethylation system of m6A (g) or m5C (h) and the efficiency. f The mRNA level of PEBP1 after transfection with indicated lentivirus. i The mRNA level of PEBP1 after transfection with indicated lentivirus. j-l RNA pulldown assay and western blot were conducted to elevate the binding capacity of protein candidates to PEBP1 mRNA. m, n The stability of GAPDH mRNA in 786-O and A-498 cells transfected with ELAVL1-shRNA was measured by real-time PCR relative to 0 h after blocking new RNA synthesis with α-amanitin. The data are presented as the mean ± SD, ***P< 0.001, n.s. = no significance. **Fig. S20**. Silence YBX1 suppresses the protein level of PEBP1. The protein levels of YBX1 and PEBP1 after transfection with indicated shRNAs. The data are presented as the mean ± SD, **P< 0.01. **Fig. S21.** Silence YBX1 promotes the mRNA decay of PEBP1. The stability of PEBP1 mRNA and GAPDH mRNA in 786-O and A-498 cells transfected with YBX1-shRNA was measured by real-time PCR relative to 0 h after blocking new RNA synthesis with α-amanitin. The data are presented as the mean ± SD, ***P< 0.001. **Fig. S22.** PEBP1P2 and m5C mediate the interaction between YBX1 and PEBP1 mRNA. a RNA pulldown assay and western blot were conducted to elevate the binding capacity of YBX1 to PEBP1 mRNA after transfection with indicated lentivirus. b MS2-RIP, RNA pulldown assay and western blot were conducted to elevate the binding capacity of YBX1 to PEBP1 mRNA after transfection with indicated shRNAs. **Fig. S23.** The protein levels of YBX1 and ELAVL1 in ccRCC and adjacent non-cancerous tissues show no significant difference. a The mRNA levels of YBX1, ELAVL1 NSUN2 and NSUN6 were analyzed according to the ccRCC dataset TCGA database. b, c IHC score of YBX1 (a) and ELAVL1 (b) in ccRCC and adjacent non-cancerous tissues. d Representative IHC images of YBX1 and ELAVL1 in ccRCC and adjacent non-cancerous tissues respectively. The data are presented as the mean ± SD, ***P< 0.001, n.s. = no significance. **Fig. S24.** The expression of potential miRNAs binding with PEBP1P2 in ccRCC. Analysis of potential miRNAs binding with PEBP1P2 in ccRCC and adjacent non-cancerous tissues were performed using TCGA data. The data are presented as the mean ± SD, **P< 0.01, ***P< 0.001. **Fig. S25.** PEBP1P2 mediates the ccRCC metastasis via sponging miR-296/-616/-3194. a-d The RNA levels of miR-296/-616/-3194 were analyzed via real-time PCR after transfection with indicate shRNAs (a), overexpressing vector (b), mimics (c) and inhibitors (d) respectively. e The miRNAs-Biotin complex were enriched by Biotin-antibody with protein extracts, and the enrichment of PEBP1P2 RNA was measured by real-time PCR. f Migration and invasion assays were conducted with transfected cells using Transwell inserts. The data are presented as the mean ± SD, ***P< 0.001. **Fig. S26.** MiR-296/-616/-3194 mediates the expression of KLF13 via direct binding. a HEK293T cells were co-transfected with indicated mRNA 3’-UTR luciferase truncations and shRNAs, and the luciferase activity was determined using a dual luciferase reporter assay after 48h. b-e the mRNA (b, c) and protein (d, e) levels of KLF13 were determined after transfection with indicated lentivirus, mimics or inhibitors. f The miRNAs-Biotin complex were enriched by Biotin-antibody with protein extracts, and the enrichment of KLF13 mRNA was measured by real-time PCR. g HEK293T cells were co-transfected with miRNA mimics and wild-type or mutant KLF13 3’-UTR luciferase reporter vector, and luciferase reporter activity was detected. The data are presented as the mean ± SD, ***P< 0.001. **Fig. S27.** MiR-296/-616/-3194 promote cell migration and invasion via inhibiting KLF13. Migration and invasion assays were conducted with transfected cells using Transwell inserts. **Fig. S28.** KLF13 is low expressed in ccRCC. a The mRNA level of KLF13 in 21 pairs of human clinical ccRCC tissues normalized to corresponding adjacent non-cancerous tissues was detected by real-time PCR. b The correlation between PEBP1P2 and KLF13 was analyzed according to the clinical ccRCC sample. c IHC score of KLF13 in ccRCC and adjacent non-cancerous tissues. d Representative IHC images of KLF13 in ccRCC and adjacent non-cancerous tissues respectively. The data are presented as the mean ± SD, *P< 0.05. **Fig. S29.** The characteristics of potential biomarker in ccRCC. a Receiver operating characteristic (ROC) analysis was constructed for quantify response prediction. b Forest plot comparing the performance of potential biomarker on OS, DSS and PFI of ccRCC. **Table S1.** The characteristics of potential prognostic indicators in ccRCC. **Table S2.** Clinical characteristics of ccRCC patients with low/high expression of *PEBP1P2.* **Table S3.** Primers used for real-time PCR. **Table S4.** Primers used for ChIP assay. **Table S5.** Primers used for RNA modification RIP-qRT-PCR analysis. **Table S6.** Probes used for RNA FISH and RNA pulldown. **Table S7.** SiRNA, shRNA and ASOs used for silencing target genes. **Table S8.** Guide RNA used for SAM system and targeted RNA methylation system. **Table S9.** Primary antibodies used in this study. **Table S10.** Small-molecule inhibitors used in this study.

## Data Availability

The datasets used and/or analyzed during the current study are available from the corresponding author upon reasonable request.
