# Supplementary material for: Low expression of PEBP1P2 promotes metastasis of clear cell renal cell carcinoma by post-transcriptional regulation of PEBP1 and KLF13 mRNA
Source: Exp Hematol Oncol. 2022 Nov 8;11:87. doi: 10.1186/s40164-022-00346-2 (PMC9644627; doi:10.1186/s40164-022-00346-2)
Supplement: Supplementary file 1 — Additional file 1: Fig. S1. Low expression of pseudogene PEBP1P2 is linked to the advanced stage of ccRCC. a-d The RNA level of PEBP1P2 was analyzed according to different group in the ccRCC dataset TCGA database. The data are presented as the mean ± SD, *P< 0.05, **P< 0.01, ***P< 0.001. Fig. S2. Low expression of pseudogene PEBP1P2 shows no effect on the progression of papillary RCC and chromophobe RCC. a-c Kaplan–Meier curve was conducted to estimate overall survival (a), disease specific survival (b) and progress free interval (c) in papillary RCC dataset of TCGA database. d-f Kaplan–Meier curve was conducted to estimate overall survival (d), disease specific survival (e) and progress free interval (f) in chromophobe RCC dataset of TCGA database. Fig. S3. The RNA level of PEBP1P2 after transfection with indicated lentivirus or ASOs. a The RNA level of PEBP1P2 after transfection with indicated shRNAs. b The RNA level of PEBP1P2 after transfection with indicated overexpressing vector. c The RNA level of PEBP1P2 after transfection with indicated lentivirus or ASOs. d The RNA level of PEBP1P2 after transfection with indicated lentivirus. The data are presented as the mean ± SD, ***P< 0.001. Fig. S4. PEBP1P2 reduces cell migration and invasion. a-c Migration and invasion assays were conducted with transfected cells using Transwell inserts. Fig. S5. Low expression of PEBP1P2 participates the formation of high migratory capacity. a Ten rounds of Transwell selection were conducted to screened out the 786-O cell with high migratory capacity (786-OHiMi) and low migratory capacity (786-OLoMi), and migration and invasion assays were conducted to confirm the construction of these two cell lines. b The RNA level of PEBP1P2 after ten rounds of Transwell selection was detected by real-time PCR. c Migration and invasion assays were conducted with transfected cells using Transwell inserts. The data are presented as the mean ± SD, ***P< 0.001. Fig. S6. The mRNA levels of 21 transcr [file 40164_2022_346_MOESM1_ESM.docx]

Supplementary Materials for

**Low expression of PEBP1P2 promotes metastasis of clear cell renal cell carcinoma by post-transcriptional regulation of PEBP1 and KLF13 mRNA**

Lei Yang^1,2†^, Haoli Yin^3†^, Yi Chen^1,2†^, Chun Pan^1,2^, Hexing Hang^1,2^, Yanwen Lu^1,2^, Wenliang Ma^1,2^, Xin Li^1,2^, Weidong Gan^3*^, Hongqian Guo^3*^, Dongmei Li^1,2*^

† These authors contributed equally to this work.

*Correspondence to: Dongmei Li: lidm@nju.edu.cn

Hongqian Guo: dr.ghq@nju.edu.cn

Weidong Gan: gwd@nju.edu.cn

**This PDF file includes:**

Supplementary Text

Figures. S1 to S29

Tables S1 to S10

Supplementary Text

**The sequence of *PEBP1P2***

GTCACACTTTAGTGGCCTGTCCTGCTCATAAACCAGCCAGACATAGCGGTGGAGACCTGTGCCCTTGGGAGGCCCCGAGCCCACATAATCGGAGAGGAGTGTGTCACTGCTGATGTCATTGCCCTTCATGTTGACCACCAGGAAATGATGCCATTCTCTGTATTTGGGATCCTTCCTGCTGGGAGCATCAGGGTCTGTCAGGACCAAGGTGTAGAGCTTCCCTGAATCAAGACCATCCCACTAAATGCTGGTGGGTCTATTCTTAACCTGGGTGGGCGTCAGCACTTTGCCCAGCTCGTCCACCACCGCCCGGGCGTAGGTGACATGCAGCAGGTGCTGTGACCGCTCGTCCATTTCTCGCAGGCTCAAGGGCCCGGACCACTTGCTGAGGTCCACCGGCATGGCAAG

Supplementary Figures


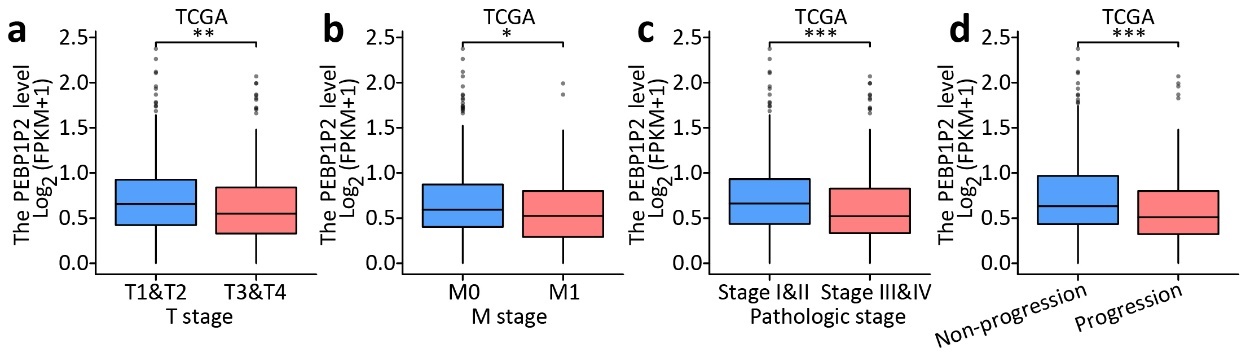


Figure. S1. Low expression of pseudogene *PEBP1P2* is linked to the advanced stage of ccRCC.

**a-d** The RNA level of *PEBP1P2* was analyzed according to different group in the ccRCC dataset TCGA database. The data are presented as the mean ± SD, **P*< 0.05, ***P*< 0.01, ****P*< 0.001


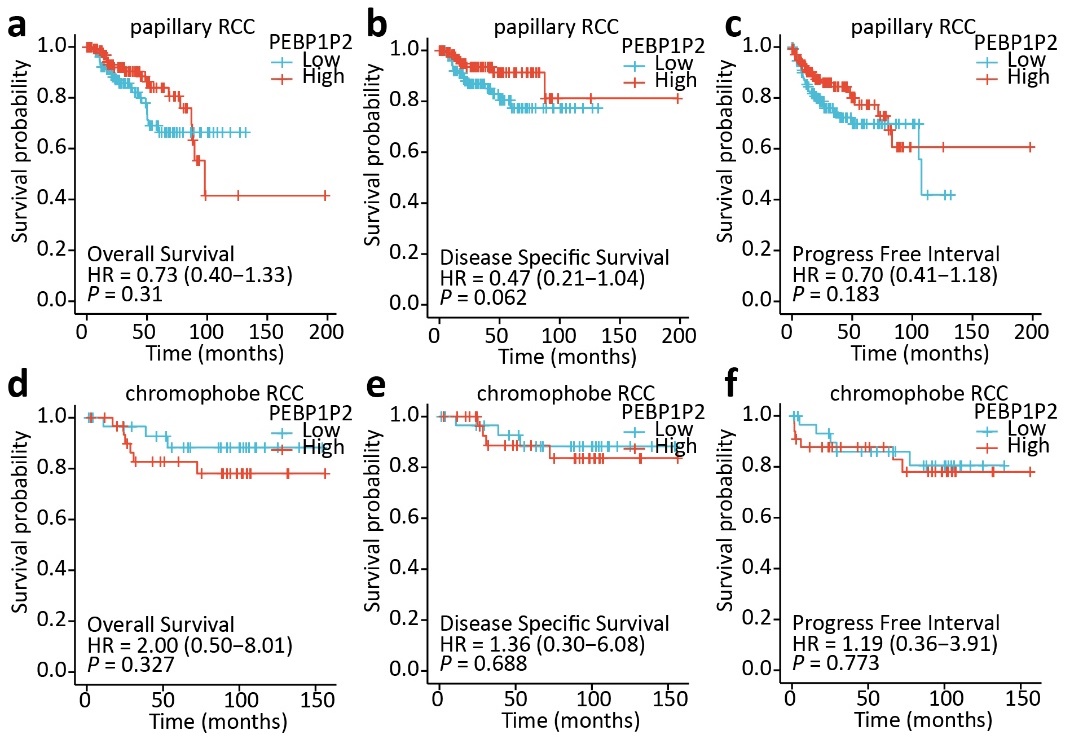


Figure. S2. Low expression of pseudogene *PEBP1P2* shows no effect on the progression of papillary RCC and chromophobe RCC.

**a-c** Kaplan–Meier curve was conducted to estimate overall survival (**a**), disease specific survival (**b**) and progress free interval (**c**) in papillary RCC dataset of TCGA database. **d-f** Kaplan–Meier curve was conducted to estimate overall survival (**d**), disease specific survival (**e**) and progress free interval (**f**) in chromophobe RCC dataset of TCGA database.


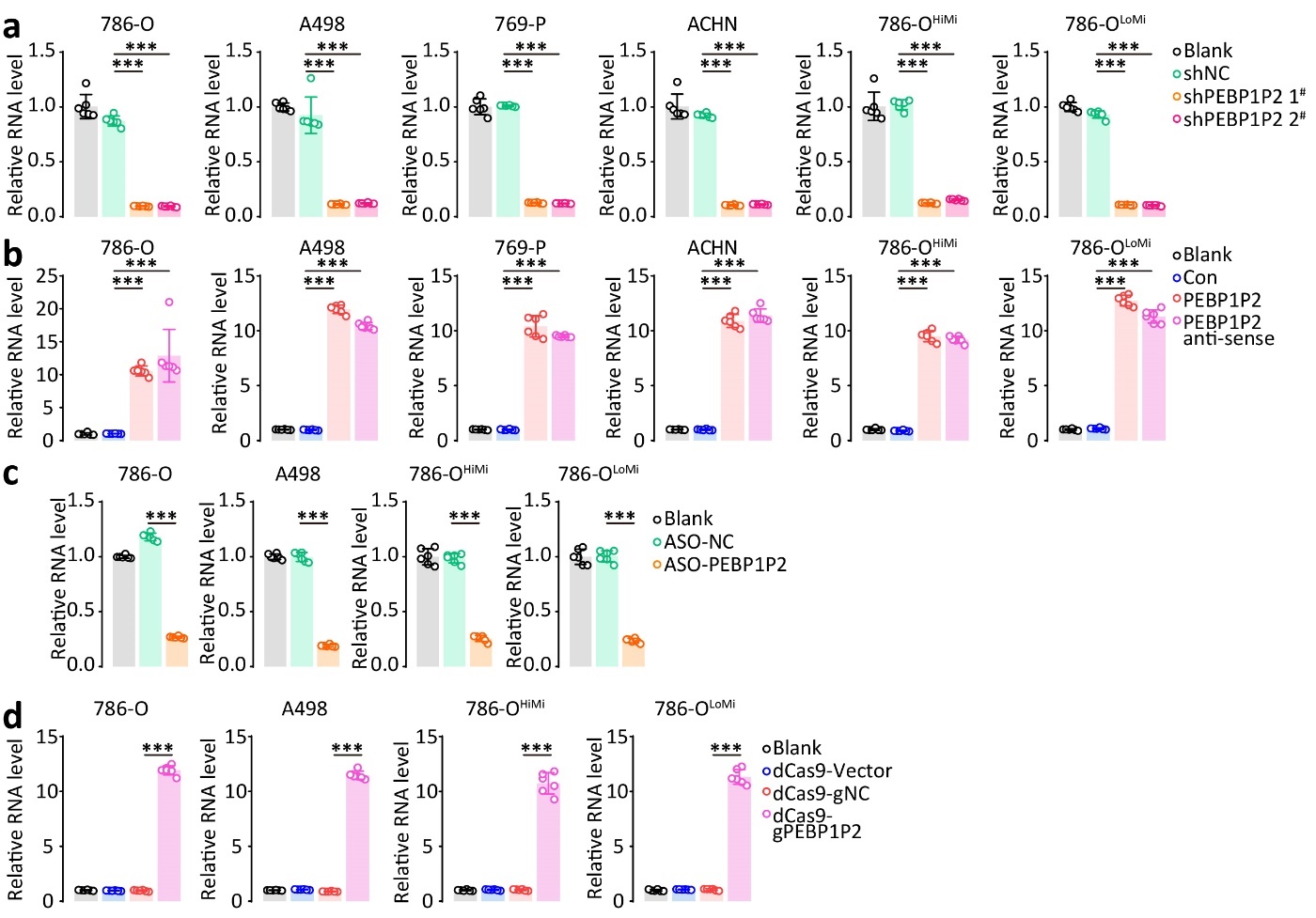


Figure. S3. The RNA level of *PEBP1P2* after transfection with indicated lentivirus or ASOs.

**a** The RNA level of *PEBP1P2* after transfection with indicated shRNAs. **b** The RNA level of *PEBP1P2* after transfection with indicated overexpressing vector. **c** The RNA level of *PEBP1P2* after transfection with indicated lentivirus or ASOs. **d** The RNA level of *PEBP1P2* after transfection with indicated lentivirus. The data are presented as the mean ± SD, ****P*< 0.001


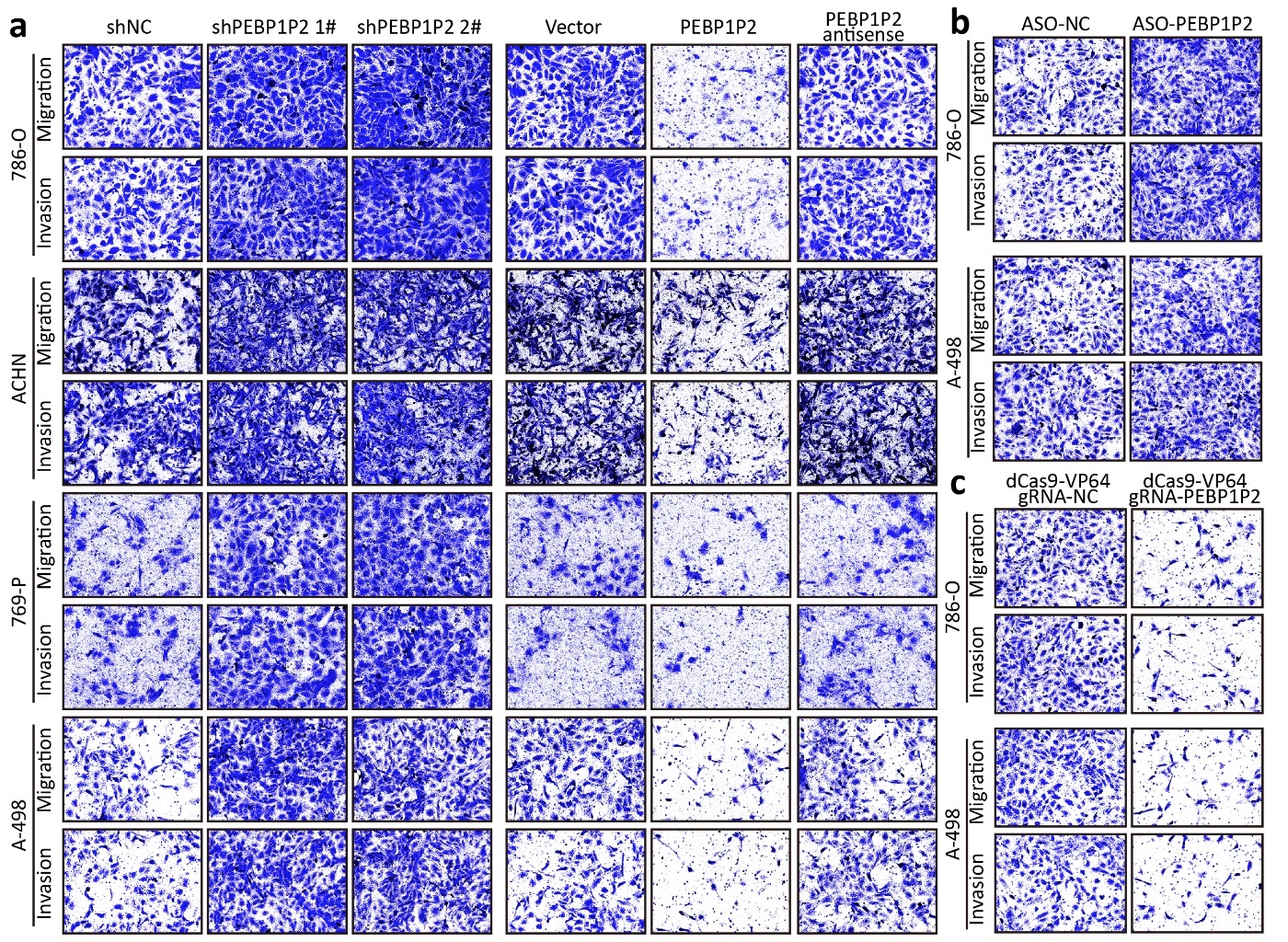


Figure. S4. *PEBP1P2* reduces cell migration and invasion.

**a-c** Migration and invasion assays were conducted with transfected cells using Transwell inserts.


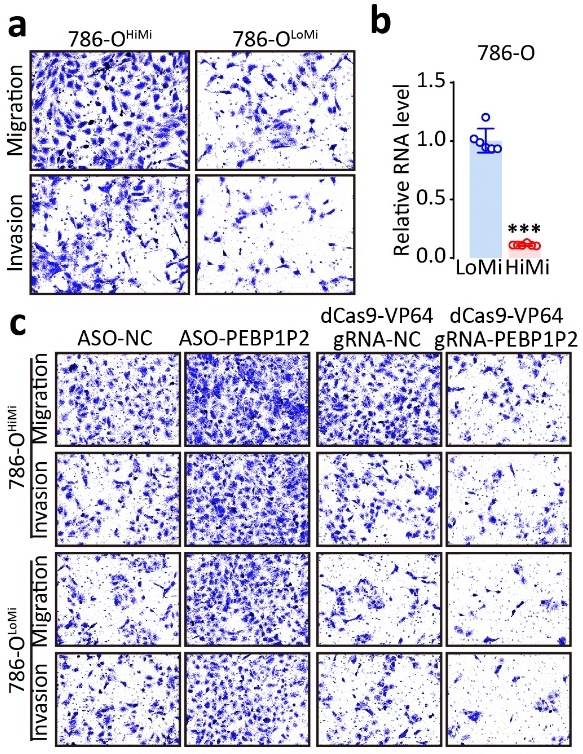


Figure. S5. Low expression of *PEBP1P2* participates the formation of high migratory capacity.

**a** Ten rounds of Transwell selection were conducted to screened out the 786-O cell with high migratory capacity (786-O^HiMi^) and low migratory capacity (786-O^LoMi^), and migration and invasion assays were conducted to confirm the construction of these two cell lines.  **b** The RNA level of *PEBP1P2* after ten rounds of Transwell selection was detected by real-time PCR. **c** Migration and invasion assays were conducted with transfected cells using Transwell inserts. The data are presented as the mean ± SD, ****P*< 0.001


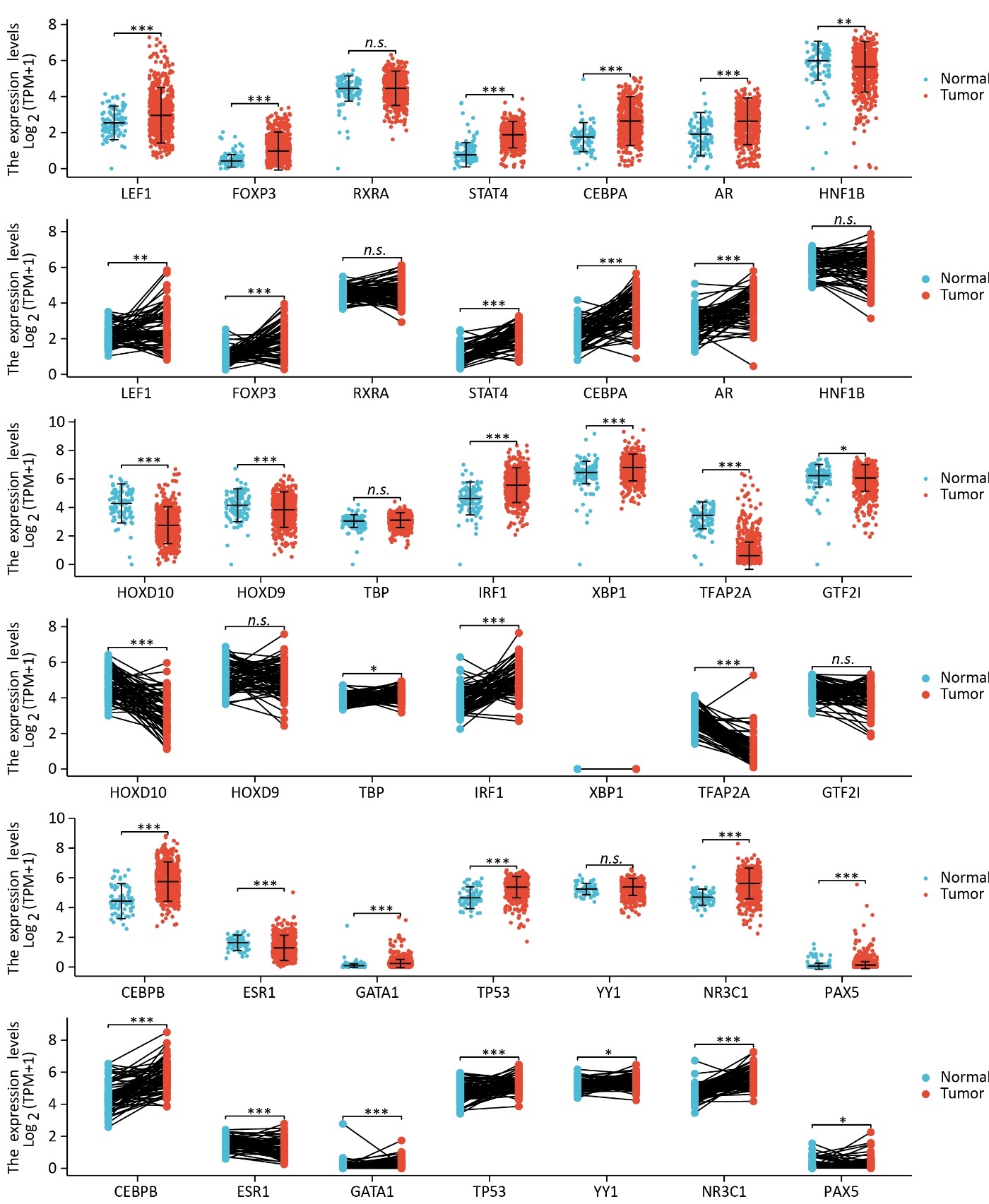


Figure. S6. The mRNA levels of 21 transcription factors were analyzed.

The mRNA level of 21 transcription factors which could bind to the promoter region of *PEBP1P2* were analyzed according to the all samples and the paired samples in the ccRCC dataset TCGA database. The data are presented as the mean ± SD, **P*< 0.05, ***P*< 0.01, ****P*< 0.001, *n.s.* = no significance


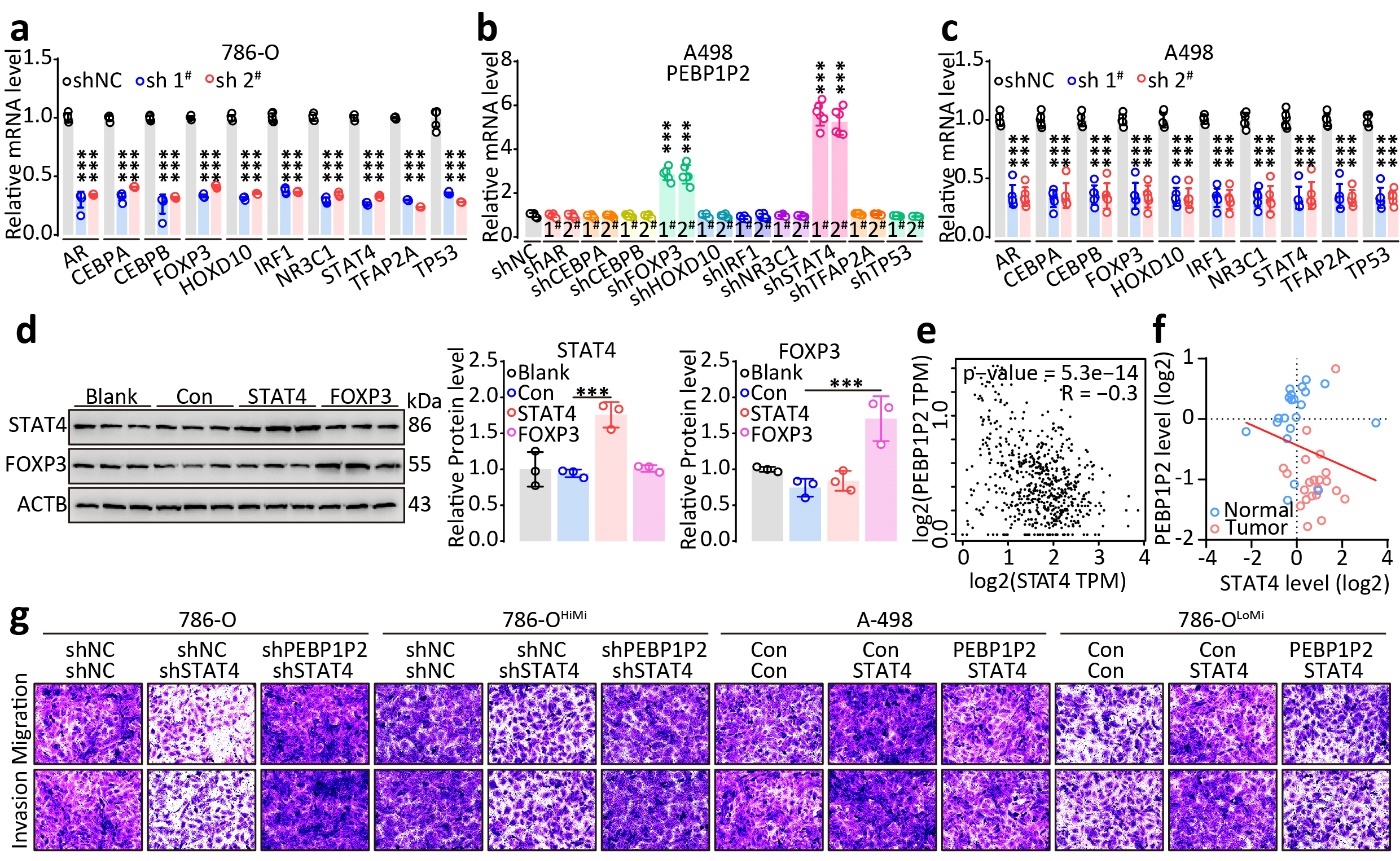


Figure. S7. The expression of *PEBP1P2* is inhibited by STAT4.

**a** The mRNA level of indicated genes after transfection with indicated shRNAs was detected by real-time PCR respectively. **b** The RNA level of *PEBP1P2* after transfection with indicated shRNAs was detected by real-time PCR. **c** The mRNA level of indicated genes after transfection with indicated shRNAs was detected by real-time PCR respectively. **d** The protein levels of STAT4 and FOXP3 were determined by western blot after overexpression of STAT4 and FOXP3. **e, f** The correlation between STAT4 and *PEBP1P2* was analyzed according to the ccRCC dataset in TCGA database (**e**) and the clinical ccRCC sample (**f**). **g** Migration and invasion assays were conducted with transfected cells using Transwell inserts. The data are presented as the mean ± SD, ****P*< 0.001


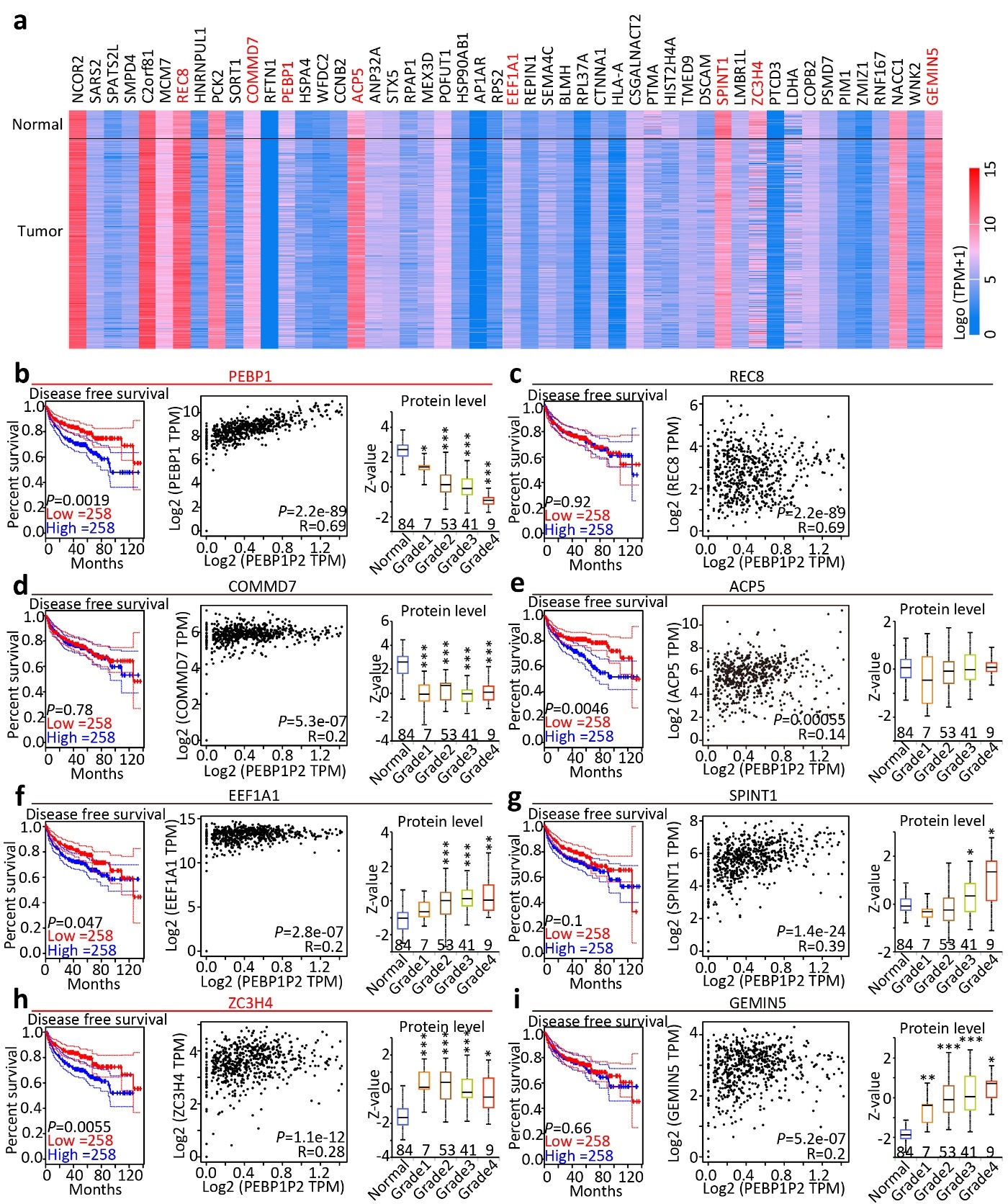


Figure. S8. The expression of potential mRNA binding with *PEBP1P2* in ccRCC.

**a** Heat map comparing the mRNA level of potential mRNA binding with *PEBP1P2* in ccRCC and adjacent non-cancerous tissues. **b-i** Analysis of potential gene in ccRCC and adjacent non-cancerous tissues were performed using TCGA data and CPTAC data. The data are presented as the mean ± SD, **P*< 0.05, ***P*< 0.01, ****P*< 0.001


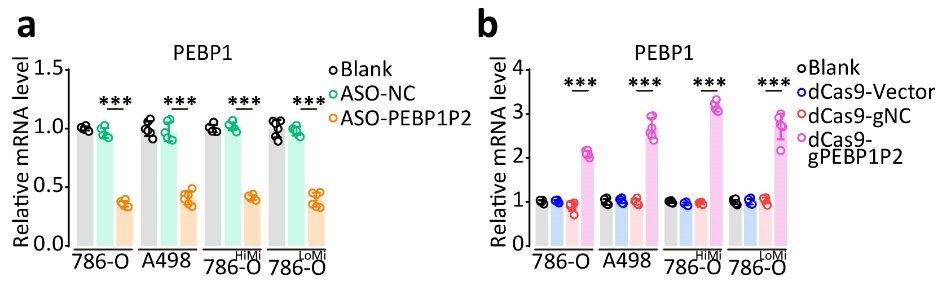


Figure. S9. The mRNA level of *PEBP1* after transfection with indicated lentivirus or ASOs.

**a** The mRNA level of *PEBP1* after transfection with indicated ASOs. **b** The mRNA level of *PEBP1* after transfection with indicated lentivirus. The data are presented as the mean ± SD, ****P*< 0.001


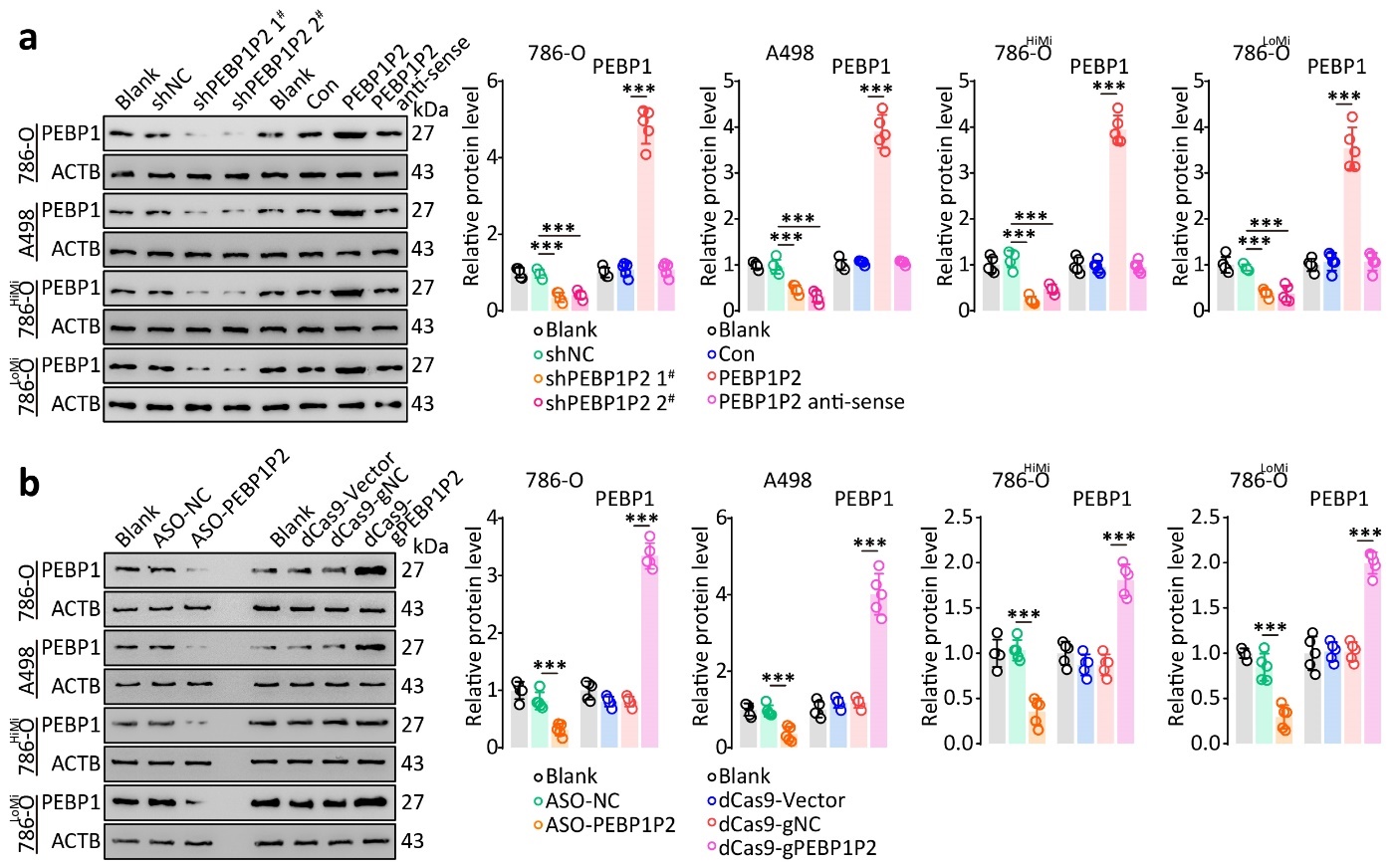


Figure. S10. The protein level of *PEBP1* after transfection with indicated lentivirus or ASOs.

**a** The mRNA level of *PEBP1* after transfection with indicated shRNAs or overexpressing vector. **b** The mRNA level of *PEBP1* after transfection with indicated ASOs or lentivirus. The data are presented as the mean ± SD, ****P*< 0.001


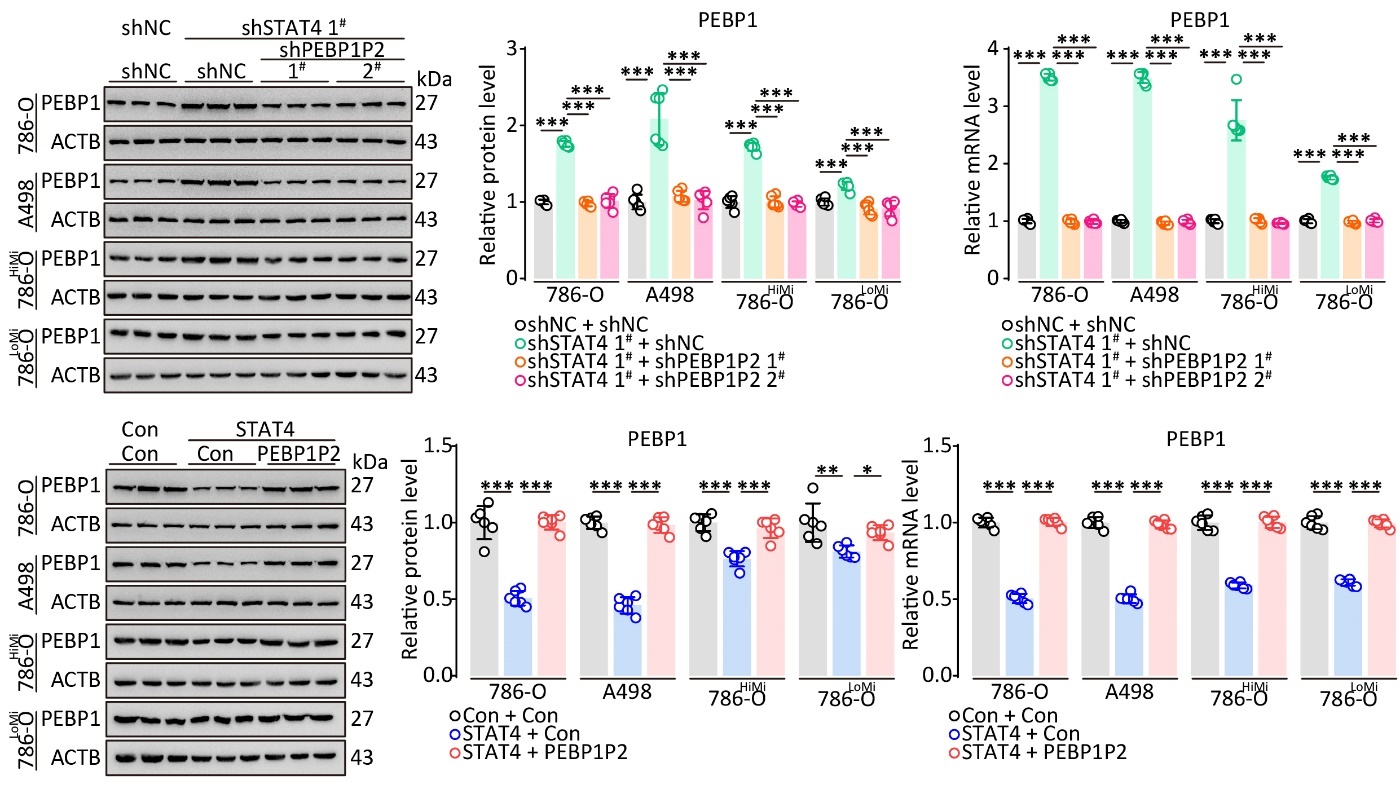


Figure. S11. The protein level of *PEBP1* after transfection with indicated lentivirus.

The protein level of *PEBP1* after transfection with indicated lentivirus. The data are presented as the mean ± SD, **P*< 0.05, ***P*< 0.01, ****P*< 0.001


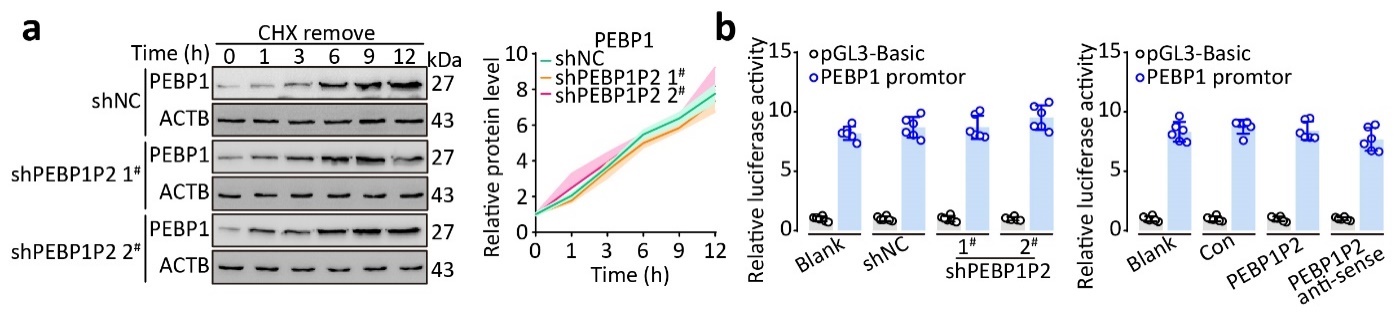


Figure. S12. *PEBP1P2* shows no influence on the transcription and translation of *PEBP1*.

**a** The protein level of *PEBP1* in cells transfected with indicated shRNAs was detected after treatment of 5 μM cycloheximide (CHX) and removing CHX for indicated time periods. **b** The luciferase activity of *PEBP1* promoter region was determined via dual luciferase assay kit after transfection with indicated shRNAs. The data are presented as the mean ± SD


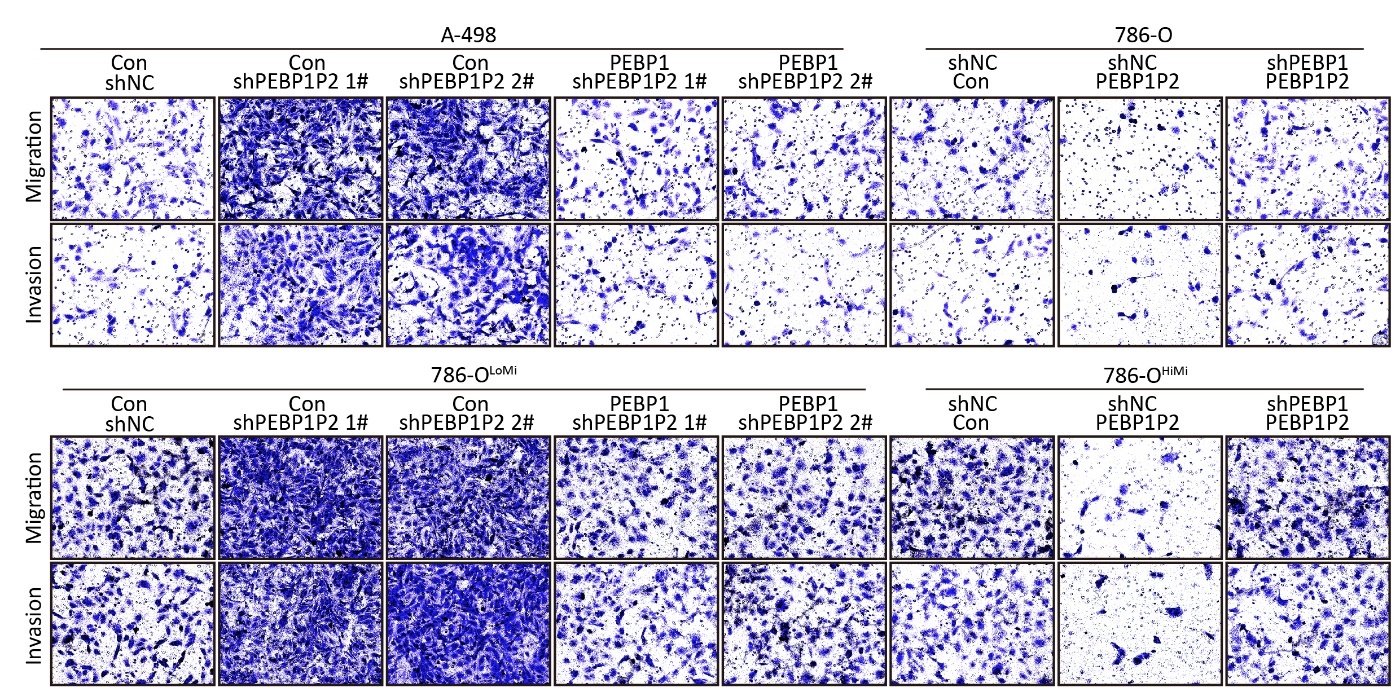


Figure. S13. *PEBP1P2* reduces cell migration and invasion via regulating the expression of *PEBP1*.

Migration and invasion assays were conducted with cells, transfected indicated lentivirus, via Transwell inserts.


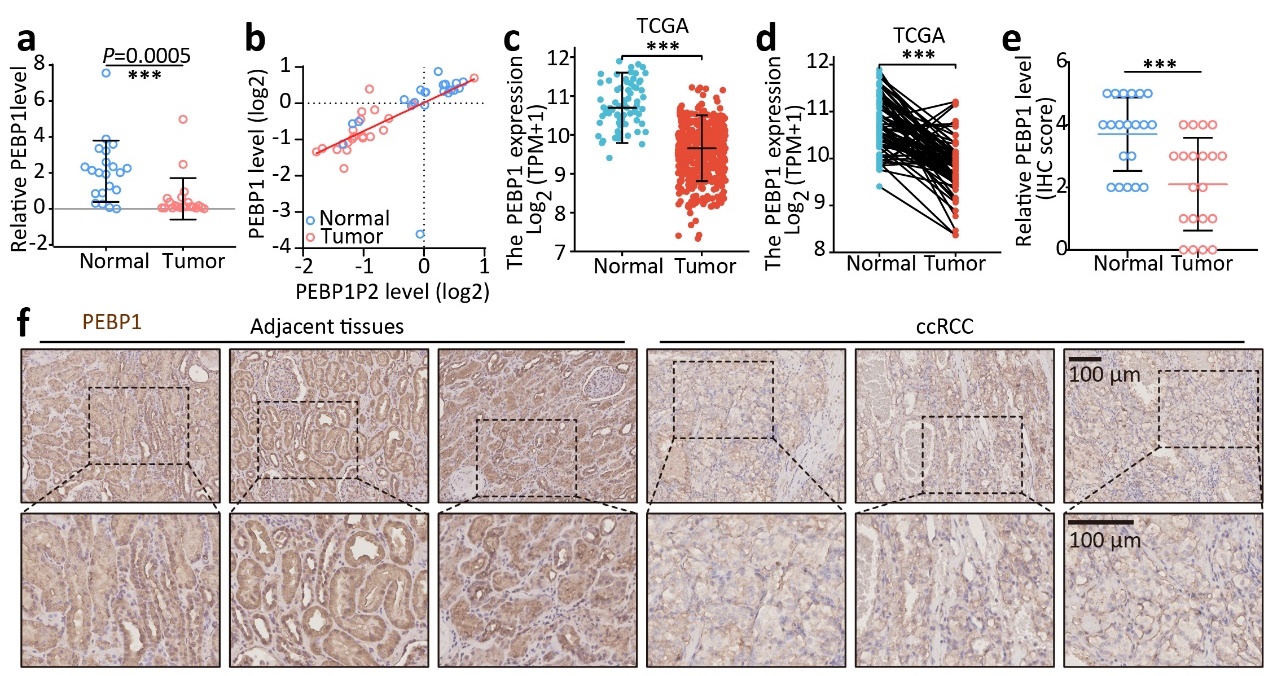


Figure. S14. *PEBP1* is low expressed in ccRCC.

**a** The mRNA level of *PEBP1* in 21 pairs of human clinical ccRCC tissues normalized to corresponding adjacent non-cancerous tissues was detected by real-time PCR. **b** The correlation between *PEBP1P2* and *PEBP1* was analyzed according to the clinical ccRCC sample. **c, d** The mRNA level of *PEBP1* was analyzed according to the all samples (**c**) and the paired samples (**d**) in the ccRCC dataset TCGA database. **e** IHC score of PEBP1 in ccRCC and adjacent non-cancerous tissues. **f** Representative IHC images of PEBP1 in ccRCC and adjacent non-cancerous tissues respectively. The data are presented as the mean ± SD, ****P*< 0.001


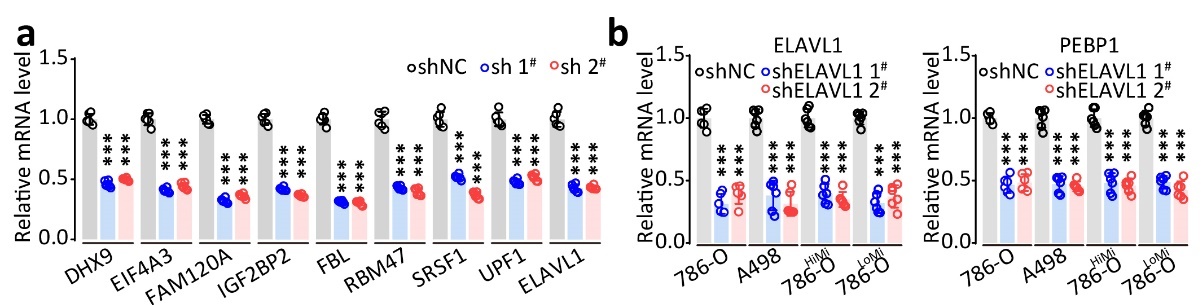


Figure. S15. Silence ELAVL1 inhibits the mRNA level of *PEBP1*.

**a** The mRNA level of indicated RNA binding proteins after transfection with indicated shRNAs. **b** The mRNA levels of *ELAVL1* and *PEBP1* after transfection with indicated shRNAs. The data are presented as the mean ± SD, ****P*< 0.001


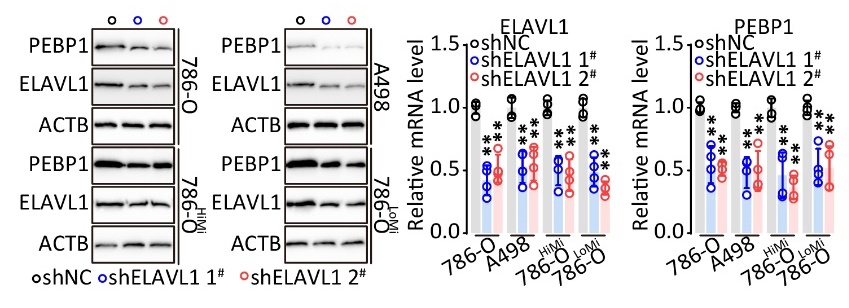


Figure. S16. Silence ELAVL1 inhibits the protein level of *PEBP1*.

The protein levels of ELAVL1 and PEBP1 after transfection with indicated shRNAs. The data are presented as the mean ± SD, ***P*< 0.01


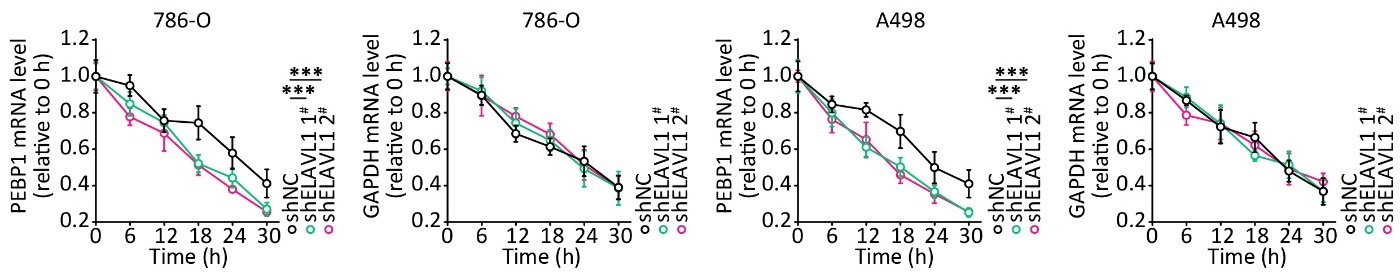


Figure. S17. Silence ELAVL1 promotes the mRNA decay of *PEBP1*.

The stability of *PEBP1* mRNA and *GAPDH* mRNA in 786-O and A-498 cells transfected with *ELAVL1*-shRNA was measured by real-time PCR relative to 0 h after blocking new RNA synthesis with α-amanitin. The data are presented as the mean ± SD, ****P*< 0.001


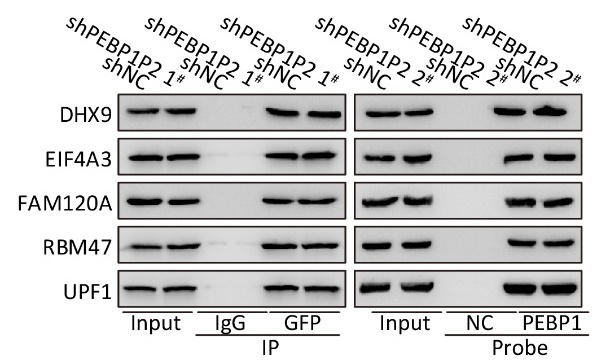


Figure. S18. Silence *PEBP1P2* shows no effect on the interaction between *PEBP1* mRNA and indicated RNA binding proteins.

MS2-RIP, RNA pulldown assay and western blot were conducted to elevate the binding capacity of protein candidates to *PEBP1* mRNA.


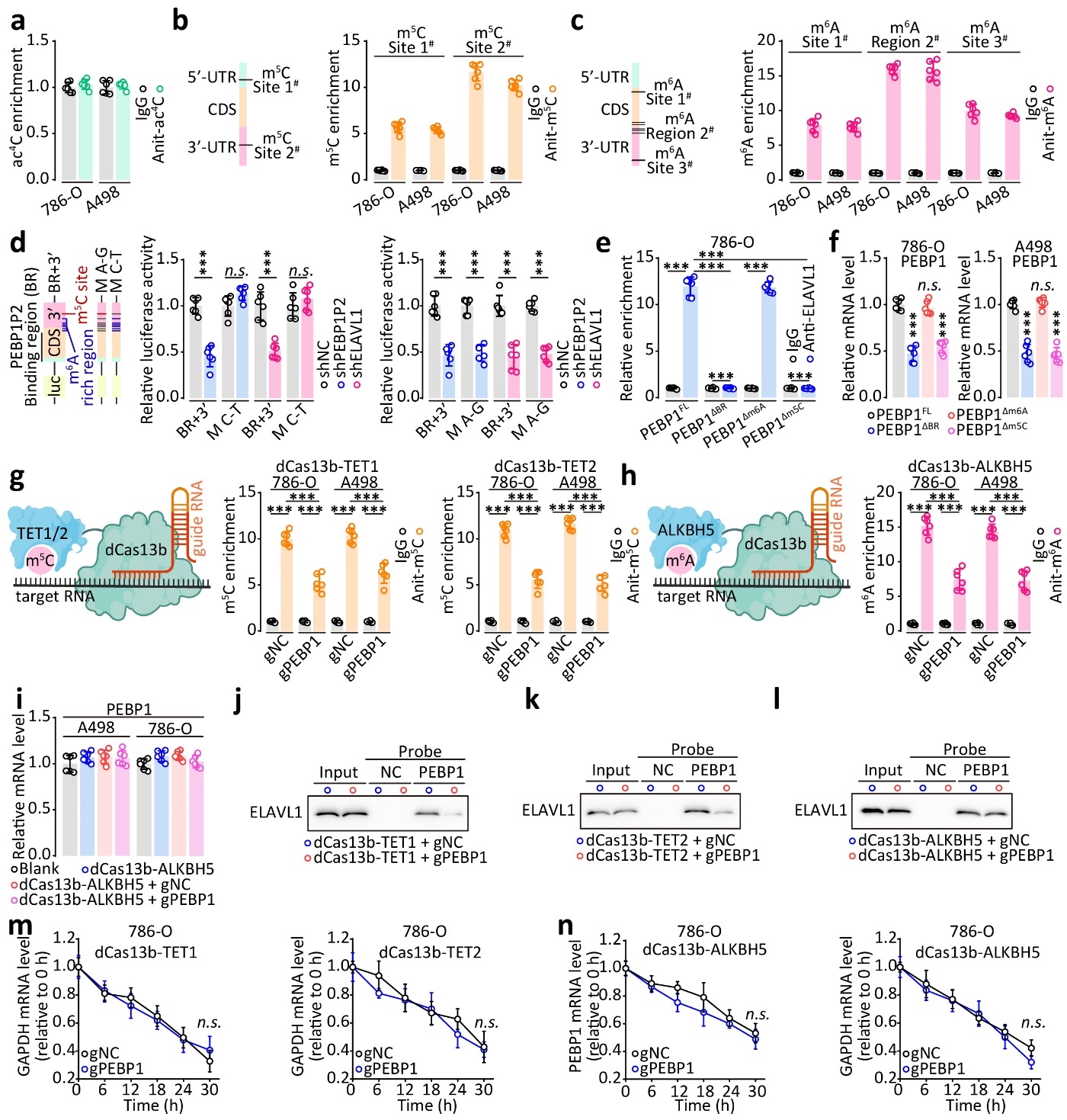


Figure. S19. *PEBP1P2* participates the stable maintenance of *PEBP1* mRNA via m^5^C.

**a-c** RNA derived from RIP assay with ac^4^C (**a**), m^5^C (**b**) and m^6^A (**c**) antibody was examined by real-time PCR. The levels of the real-time PCR products were normalized relative to IgG control. **d** Luciferase activity of *PEBP1*-wild-type and the *PEBP1*-mutation was measured after transfected with indicated lentivirus. **e** RNA derived from RIP assay with ELAVL1 antibody was examined by real-time PCR. The levels of the real-time PCR products were normalized relative to IgG control. **g, h** Schematic illustration of targeted RNA demethylation system of m^6^A (**g**) or m^5^C (**h**) and the efficiency. **f** The mRNA level of *PEBP1* after transfection with indicated lentivirus. **i** The mRNA level of *PEBP1* after transfection with indicated lentivirus. **j-l** RNA pulldown assay and western blot were conducted to elevate the binding capacity of protein candidates to *PEBP1* mRNA. **m, n** The stability of *GAPDH* mRNA in 786-O and A-498 cells transfected with *ELAVL1*-shRNA was measured by real-time PCR relative to 0 h after blocking new RNA synthesis with α-amanitin. The data are presented as the mean ± SD, ****P*< 0.001, *n.s.* = no significance


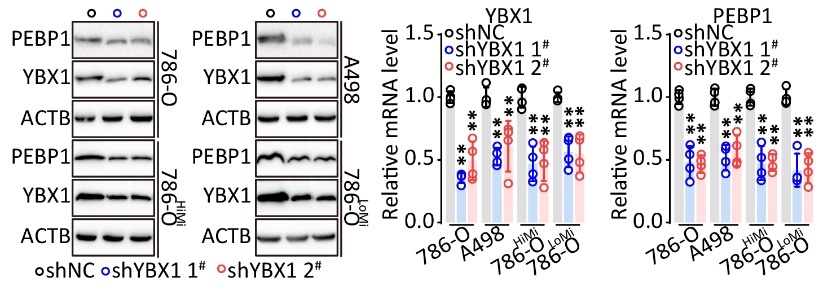


Figure. S20. Silence YBX1 suppresses the protein level of *PEBP1*.

The protein levels of YBX1 and PEBP1 after transfection with indicated shRNAs. The data are presented as the mean ± SD, ***P*< 0.01


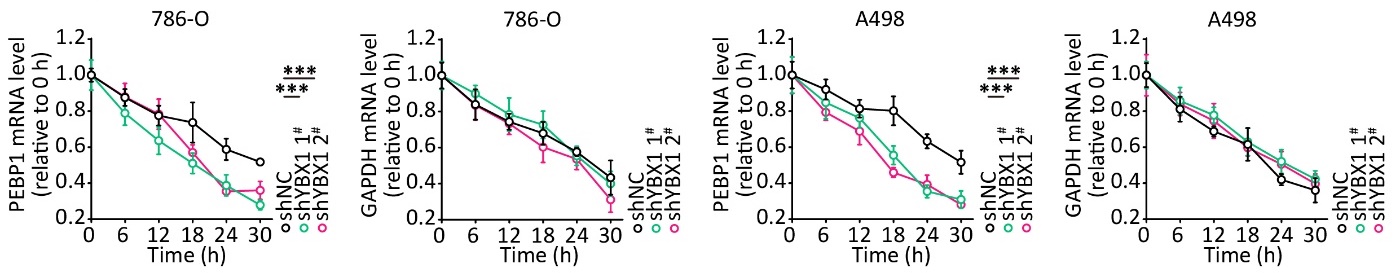


Figure. S21. Silence YBX1 promotes the mRNA decay of *PEBP1*.

The stability of *PEBP1* mRNA and *GAPDH* mRNA in 786-O and A-498 cells transfected with *YBX1*-shRNA was measured by real-time PCR relative to 0 h after blocking new RNA synthesis with α-amanitin. The data are presented as the mean ± SD, ****P*< 0.001


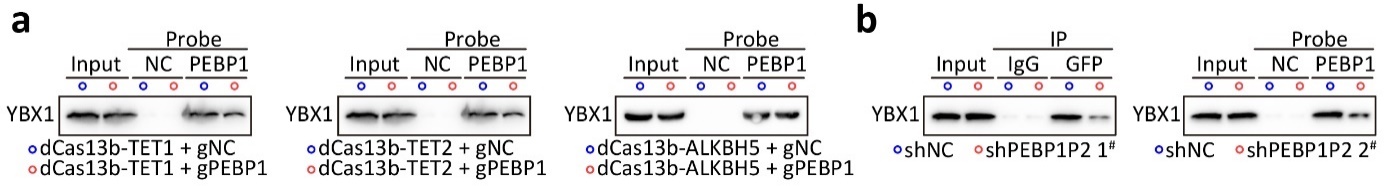


Figure. S22. *PEBP1P2* and m^5^C mediate the interaction between YBX1 and *PEBP1* mRNA.

**a** RNA pulldown assay and western blot were conducted to elevate the binding capacity of YBX1 to *PEBP1* mRNA after transfection with indicated lentivirus. **b** MS2-RIP, RNA pulldown assay and western blot were conducted to elevate the binding capacity of YBX1 to *PEBP1* mRNA after transfection with indicated shRNAs.


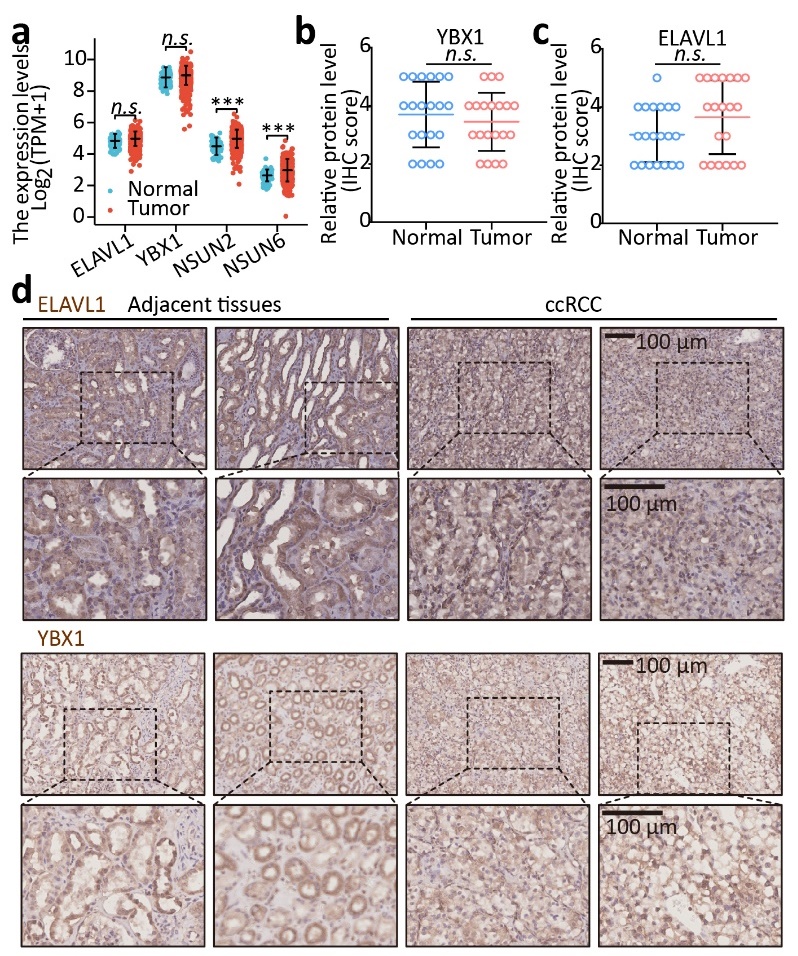


Figure. S23. The protein levels of YBX1 and ELAVL1 in ccRCC and adjacent non-cancerous tissues show no significant difference.

**a** The mRNA levels of *YBX1, ELAVL1 NSUN2* and *NSUN6* were analyzed according to the ccRCC dataset TCGA database. **b, c** IHC score of YBX1 (**a**) and ELAVL1 (**b**) in ccRCC and adjacent non-cancerous tissues. **d** Representative IHC images of YBX1 and ELAVL1 in ccRCC and adjacent non-cancerous tissues respectively. The data are presented as the mean ± SD, ****P*< 0.001, *n.s.* = no significance


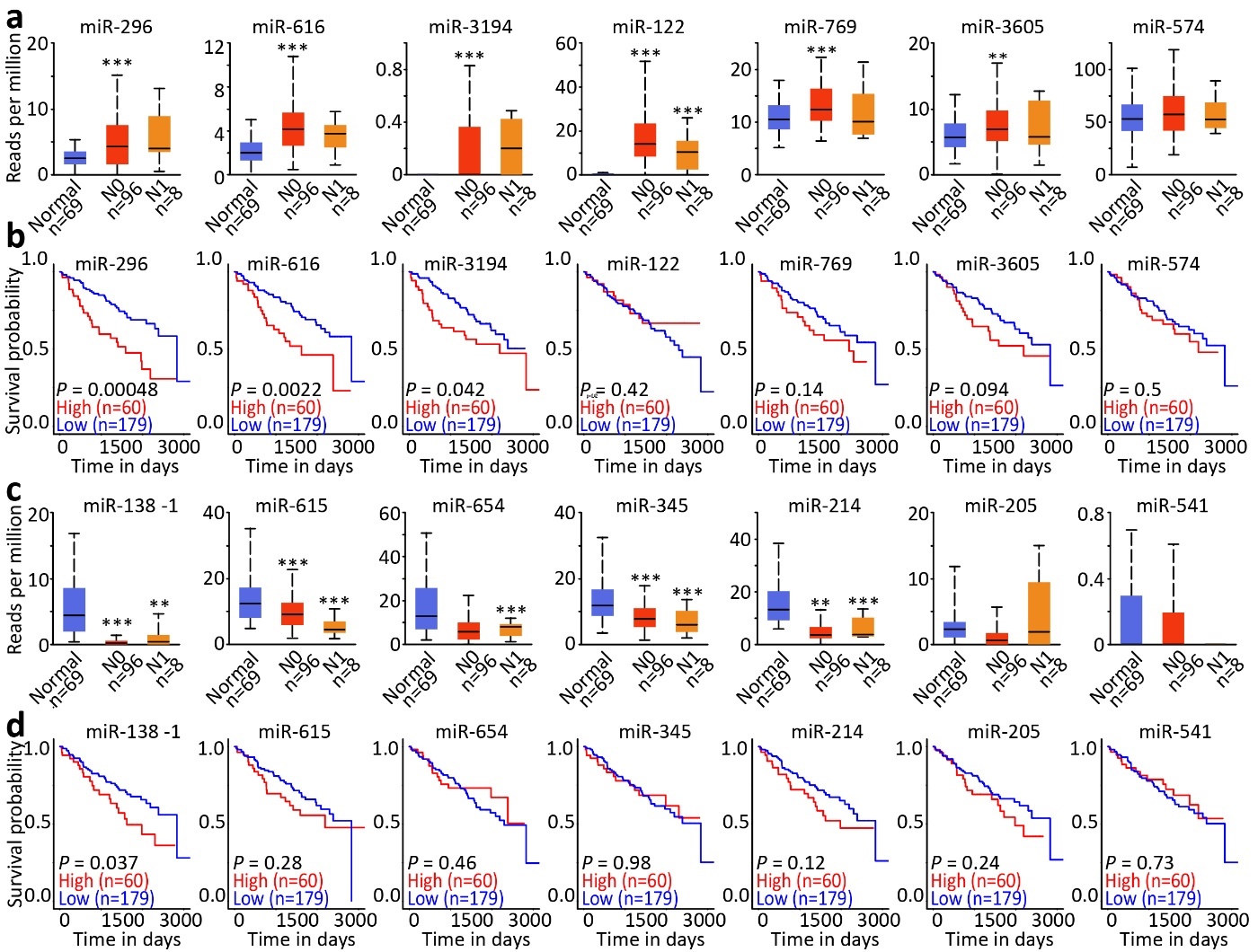


Figure. S24. The expression of potential miRNAs binding with *PEBP1P2* in ccRCC.

Analysis of potential miRNAs binding with *PEBP1P2* in ccRCC and adjacent non-cancerous tissues were performed using TCGA data. The data are presented as the mean ± SD, ***P*< 0.01, ****P*< 0.001


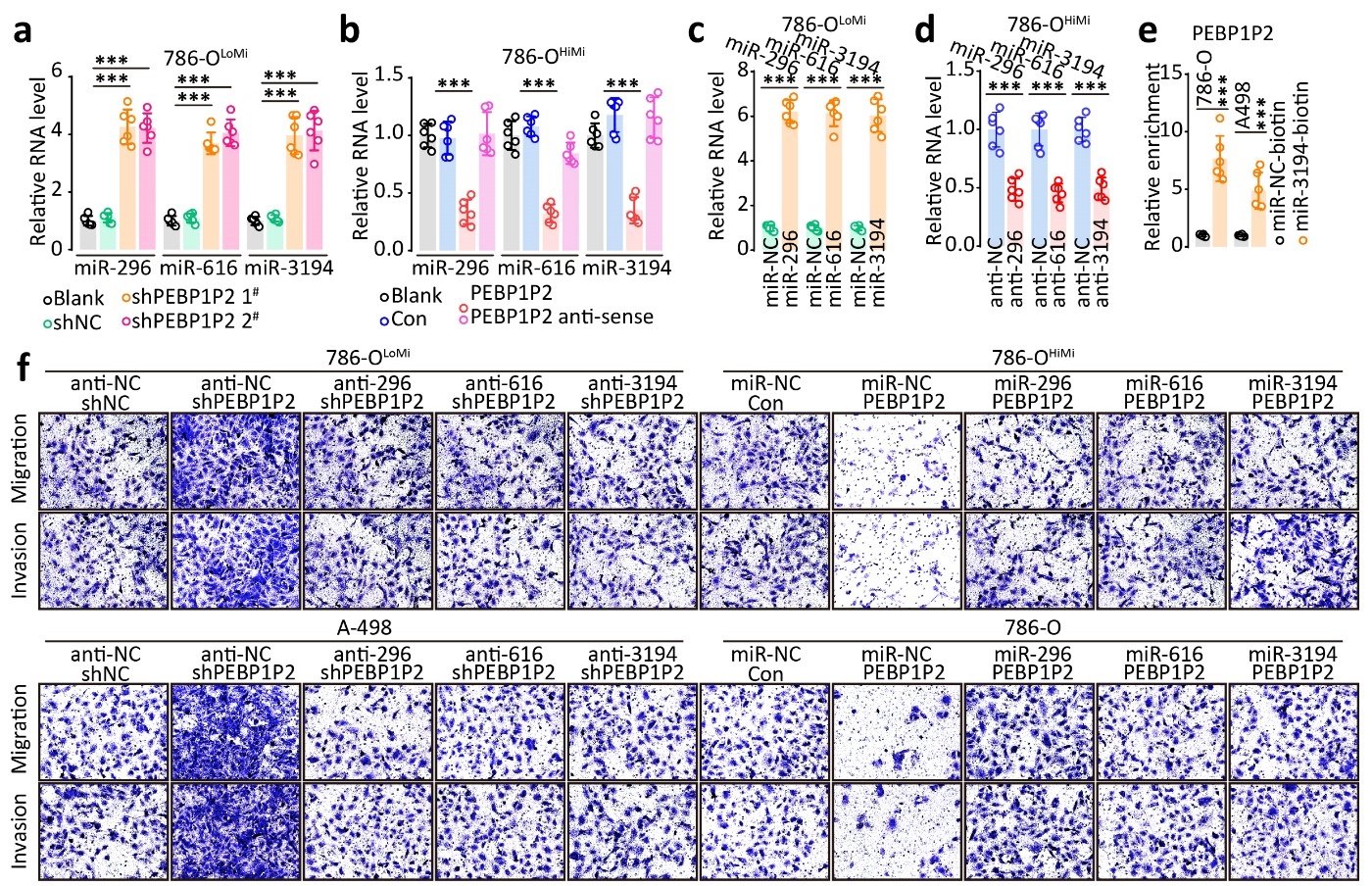


Figure. S25. *PEBP1P2* mediates the ccRCC metastasis via sponging miR-296/-616/-3194.

**a-d** The RNA levels of miR-296/-616/-3194 were analyzed via real-time PCR after transfection with indicate shRNAs (**a**), overexpressing vector (**b**), mimics (**c**) and inhibitors (**d**) respectively. **e** The miRNAs-Biotin complex were enriched by Biotin-antibody with protein extracts, and the enrichment of *PEBP1P2* RNA was measured by real-time PCR. **f** Migration and invasion assays were conducted with transfected cells using Transwell inserts. The data are presented as the mean ± SD, ****P*< 0.001


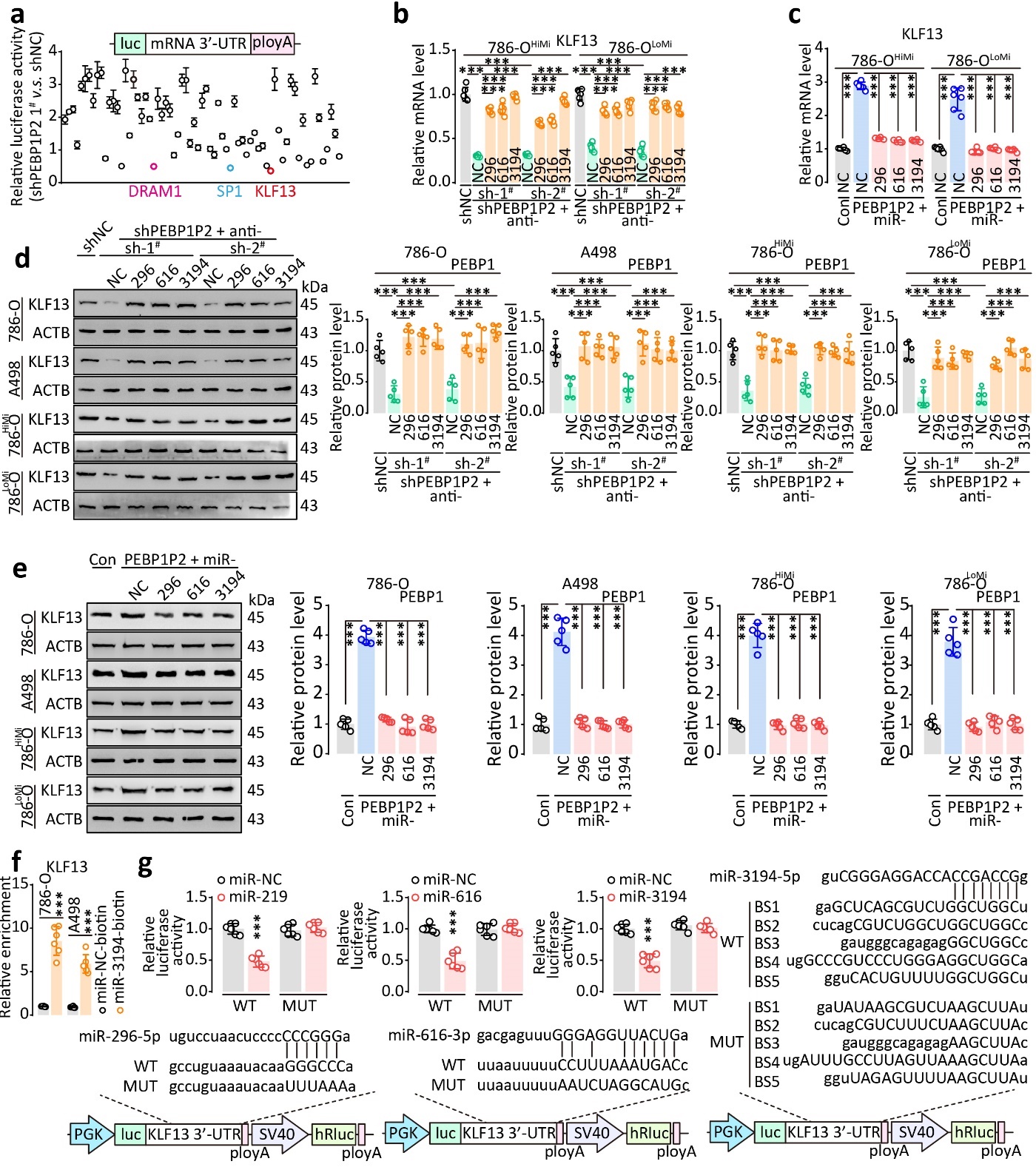


Figure. S26. MiR-296/-616/-3194 mediates the expression of *KLF13* via direct binding.

**a** HEK293T cells were co-transfected with indicated mRNA 3’-UTR luciferase truncations and shRNAs, and the luciferase activity was determined using a dual luciferase reporter assay after 48h. **b-e** the mRNA (**b, c**) and protein (**d, e**) levels of *KLF13* were determined after transfection with indicated lentivirus, mimics or inhibitors. **f** The miRNAs-Biotin complex were enriched by Biotin-antibody with protein extracts, and the enrichment of *KLF13* mRNA was measured by real-time PCR. **g** HEK293T cells were co-transfected with miRNA mimics and wild-type or mutant *KLF13* 3’-UTR luciferase reporter vector, and luciferase reporter activity was detected. The data are presented as the mean ± SD, ****P*< 0.001


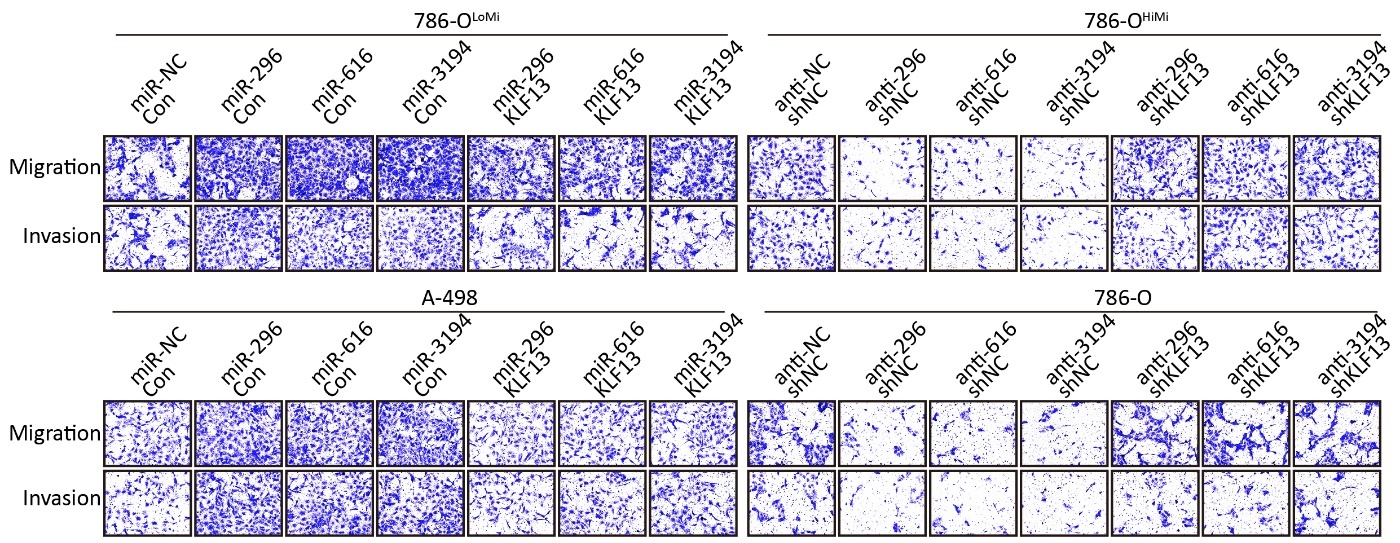


Figure. S27. MiR-296/-616/-3194 promote cell migration and invasion via inhibiting *KLF13*.

Migration and invasion assays were conducted with transfected cells using Transwell inserts.


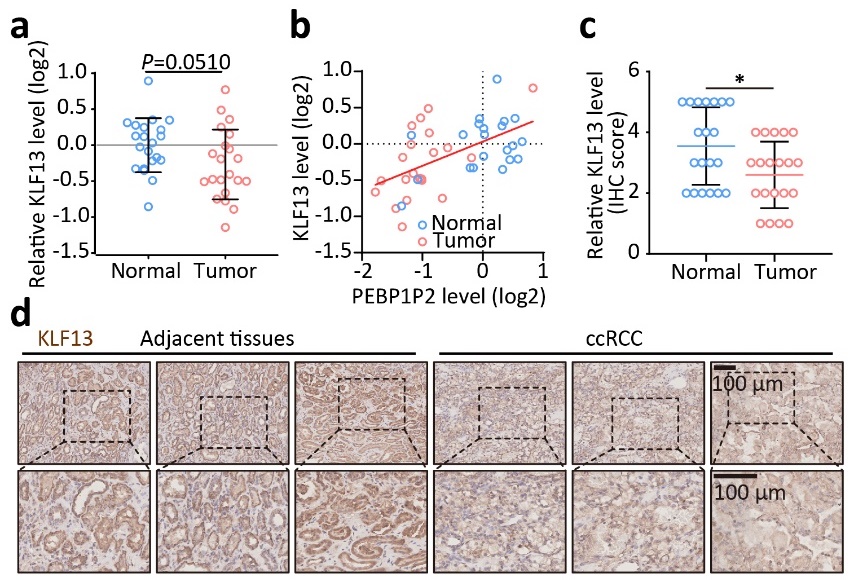


Figure. S28. *KLF13* is low expressed in ccRCC.

**a** The mRNA level of *KLF13* in 21 pairs of human clinical ccRCC tissues normalized to corresponding adjacent non-cancerous tissues was detected by real-time PCR. **b** The correlation between *PEBP1P2* and *KLF13* was analyzed according to the clinical ccRCC sample. **c** IHC score of KLF13 in ccRCC and adjacent non-cancerous tissues. **d** Representative IHC images of KLF13 in ccRCC and adjacent non-cancerous tissues respectively. The data are presented as the mean ± SD, **P*< 0.05


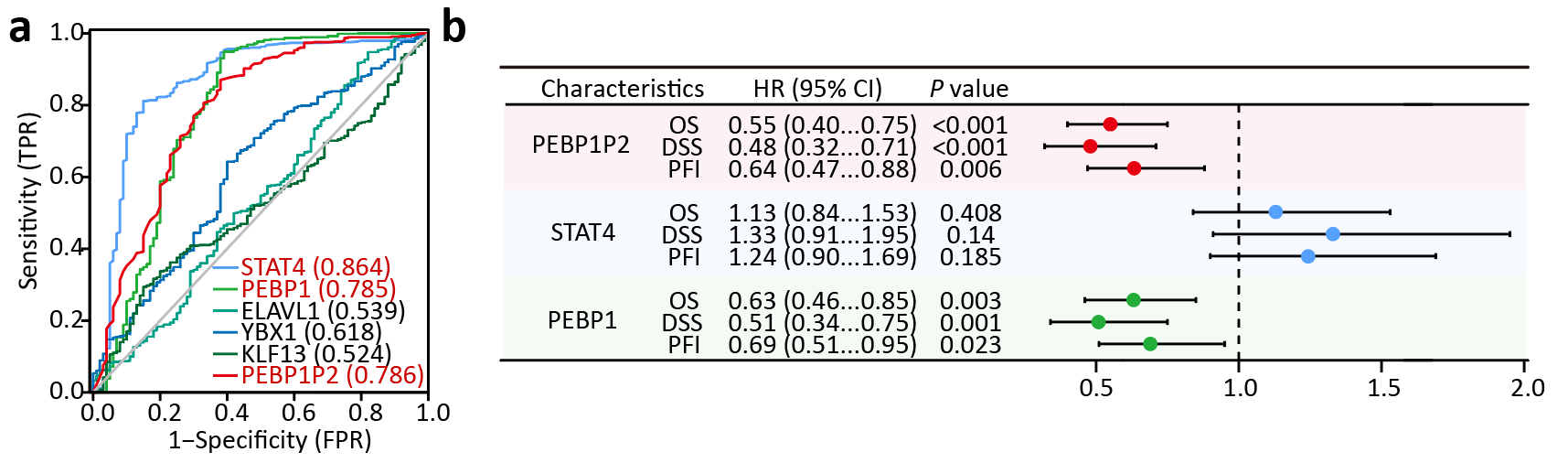


Figure. S29. The characteristics of potential biomarker in ccRCC.

**a** Receiver operating characteristic (ROC) analysis was constructed for quantify response prediction. **b** Forest plot comparing the performance of potential biomarker on OS, DSS and PFI of ccRCC.

Supplementary Tables

Table S1. The characteristics of potential prognostic indicators in ccRCC.

| **Gene Symbol** | **Gene ID** | **P-Value (Survival os)** |
| --- | --- | --- |
| [PEBP1P2](http://gepia.cancer-pku.cn/detail.php?gene=PEBP1P2) | ENSG00000270532.1 | 1.85E-14 |
| [RP11-379F4.8](http://gepia.cancer-pku.cn/detail.php?gene=RP11-379F4.8) | ENSG00000271778.1 | 3.42E-14 |
| [SOWAHB](http://gepia.cancer-pku.cn/detail.php?gene=SOWAHB) | ENSG00000186212.3 | 1.84E-13 |
| [CPT2](http://gepia.cancer-pku.cn/detail.php?gene=CPT2) | ENSG00000157184.5 | 1.92E-13 |
| [WDR72](http://gepia.cancer-pku.cn/detail.php?gene=WDR72) | ENSG00000166415.14 | 2.25E-13 |
| [DHRS12](http://gepia.cancer-pku.cn/detail.php?gene=DHRS12) | ENSG00000102796.10 | 1.30E-12 |
| [TOLLIP](http://gepia.cancer-pku.cn/detail.php?gene=TOLLIP) | ENSG00000078902.15 | 1.44E-12 |
| [TPRG1L](http://gepia.cancer-pku.cn/detail.php?gene=TPRG1L) | ENSG00000158109.14 | 1.59E-12 |
| [BAG1](http://gepia.cancer-pku.cn/detail.php?gene=BAG1) | ENSG00000107262.16 | 3.58E-12 |
| [SLC1A1](http://gepia.cancer-pku.cn/detail.php?gene=SLC1A1) | ENSG00000106688.11 | 5.68E-12 |
| [DDAH1](http://gepia.cancer-pku.cn/detail.php?gene=DDAH1) | ENSG00000153904.18 | 7.01E-12 |
| [DMGDH](http://gepia.cancer-pku.cn/detail.php?gene=DMGDH) | ENSG00000132837.14 | 7.59E-12 |
| [C1orf210](http://gepia.cancer-pku.cn/detail.php?gene=C1orf210) | ENSG00000253313.5 | 7.72E-12 |
| [IL20RB](http://gepia.cancer-pku.cn/detail.php?gene=IL20RB) | ENSG00000174564.12 | 7.83E-12 |
| [FBXO3](http://gepia.cancer-pku.cn/detail.php?gene=FBXO3) | ENSG00000110429.13 | 1.17E-11 |
| [FZD1](http://gepia.cancer-pku.cn/detail.php?gene=FZD1) | ENSG00000157240.3 | 1.48E-11 |
| [PAIP2B](http://gepia.cancer-pku.cn/detail.php?gene=PAIP2B) | ENSG00000124374.8 | 1.51E-11 |
| [CYP3A7](http://gepia.cancer-pku.cn/detail.php?gene=CYP3A7) | ENSG00000160870.12 | 1.72E-11 |
| [CRB3](http://gepia.cancer-pku.cn/detail.php?gene=CRB3) | ENSG00000130545.15 | 1.80E-11 |
| [KL](http://gepia.cancer-pku.cn/detail.php?gene=KL) | ENSG00000133116.7 | 1.87E-11 |
| [MPP7](http://gepia.cancer-pku.cn/detail.php?gene=MPP7) | ENSG00000150054.18 | 2.05E-11 |
| [GIPC2](http://gepia.cancer-pku.cn/detail.php?gene=GIPC2) | ENSG00000137960.5 | 2.07E-11 |
| [TMEM38B](http://gepia.cancer-pku.cn/detail.php?gene=TMEM38B) | ENSG00000095209.11 | 2.09E-11 |
| [SORBS2](http://gepia.cancer-pku.cn/detail.php?gene=SORBS2) | ENSG00000154556.17 | 2.59E-11 |
| [RP11-343C2.11](http://gepia.cancer-pku.cn/detail.php?gene=RP11-343C2.11) | ENSG00000260914.3 | 2.72E-11 |
| [NDUFB6](http://gepia.cancer-pku.cn/detail.php?gene=NDUFB6) | ENSG00000165264.10 | 3.37E-11 |
| [PANK1](http://gepia.cancer-pku.cn/detail.php?gene=PANK1) | ENSG00000152782.16 | 3.41E-11 |
| [SLC16A12](http://gepia.cancer-pku.cn/detail.php?gene=SLC16A12) | ENSG00000152779.13 | 3.52E-11 |
| [BBOX1](http://gepia.cancer-pku.cn/detail.php?gene=BBOX1) | ENSG00000129151.8 | 4.23E-11 |
| [RETSAT](http://gepia.cancer-pku.cn/detail.php?gene=RETSAT) | ENSG00000042445.13 | 4.25E-11 |
| [HNMT](http://gepia.cancer-pku.cn/detail.php?gene=HNMT) | ENSG00000150540.13 | 4.25E-11 |
| [RP11-407N17.3](http://gepia.cancer-pku.cn/detail.php?gene=RP11-407N17.3) | ENSG00000258941.3 | 4.59E-11 |
| [DMRTA1](http://gepia.cancer-pku.cn/detail.php?gene=DMRTA1) | ENSG00000176399.3 | 5.41E-11 |
| [ANK3](http://gepia.cancer-pku.cn/detail.php?gene=ANK3) | ENSG00000151150.20 | 5.66E-11 |
| [CRYL1](http://gepia.cancer-pku.cn/detail.php?gene=CRYL1) | ENSG00000165475.13 | 5.94E-11 |
| [C11orf54](http://gepia.cancer-pku.cn/detail.php?gene=C11orf54) | ENSG00000182919.14 | 7.17E-11 |
| [G6PC](http://gepia.cancer-pku.cn/detail.php?gene=G6PC) | ENSG00000131482.9 | 7.58E-11 |
| [SPATA18](http://gepia.cancer-pku.cn/detail.php?gene=SPATA18) | ENSG00000163071.10 | 7.82E-11 |
| [SOX6](http://gepia.cancer-pku.cn/detail.php?gene=SOX6) | ENSG00000110693.15 | 8.75E-11 |
| [RP11-416A14.1](http://gepia.cancer-pku.cn/detail.php?gene=RP11-416A14.1) | ENSG00000270040.1 | 8.91E-11 |
| [HIBCH](http://gepia.cancer-pku.cn/detail.php?gene=HIBCH) | ENSG00000198130.14 | 9.55E-11 |
| [GALK2](http://gepia.cancer-pku.cn/detail.php?gene=GALK2) | ENSG00000156958.14 | 1.02E-10 |
| [MRPL49](http://gepia.cancer-pku.cn/detail.php?gene=MRPL49) | ENSG00000149792.8 | 1.04E-10 |
| [COL7A1](http://gepia.cancer-pku.cn/detail.php?gene=COL7A1) | ENSG00000114270.15 | 1.21E-10 |
| [TLN2](http://gepia.cancer-pku.cn/detail.php?gene=TLN2) | ENSG00000171914.14 | 1.25E-10 |
| [NCR3LG1](http://gepia.cancer-pku.cn/detail.php?gene=NCR3LG1) | ENSG00000188211.8 | 1.46E-10 |
| [TMEM126B](http://gepia.cancer-pku.cn/detail.php?gene=TMEM126B) | ENSG00000171204.12 | 1.50E-10 |
| [SNX30](http://gepia.cancer-pku.cn/detail.php?gene=SNX30) | ENSG00000148158.16 | 1.50E-10 |
| [MOB3B](http://gepia.cancer-pku.cn/detail.php?gene=MOB3B) | ENSG00000120162.9 | 1.58E-10 |
| [ACADM](http://gepia.cancer-pku.cn/detail.php?gene=ACADM) | ENSG00000117054.13 | 1.76E-10 |
| [PAFAH2](http://gepia.cancer-pku.cn/detail.php?gene=PAFAH2) | ENSG00000158006.13 | 1.79E-10 |
| [ALDH6A1](http://gepia.cancer-pku.cn/detail.php?gene=ALDH6A1) | ENSG00000119711.12 | 1.81E-10 |
| [ASTN2](http://gepia.cancer-pku.cn/detail.php?gene=ASTN2) | ENSG00000148219.16 | 1.82E-10 |
| [SAMM50](http://gepia.cancer-pku.cn/detail.php?gene=SAMM50) | ENSG00000100347.14 | 1.94E-10 |
| [ZNF658](http://gepia.cancer-pku.cn/detail.php?gene=ZNF658) | ENSG00000274349.4 | 2.13E-10 |
| [HS1BP3](http://gepia.cancer-pku.cn/detail.php?gene=HS1BP3) | ENSG00000118960.12 | 2.32E-10 |
| [RP11-626G11.3](http://gepia.cancer-pku.cn/detail.php?gene=RP11-626G11.3) | ENSG00000261759.1 | 2.44E-10 |
| [CYP3A4](http://gepia.cancer-pku.cn/detail.php?gene=CYP3A4) | ENSG00000160868.14 | 2.51E-10 |
| [ENPP5](http://gepia.cancer-pku.cn/detail.php?gene=ENPP5) | ENSG00000112796.9 | 2.61E-10 |
| [SH3BGRL2](http://gepia.cancer-pku.cn/detail.php?gene=SH3BGRL2) | ENSG00000198478.7 | 2.63E-10 |
| [ATP6V1G1](http://gepia.cancer-pku.cn/detail.php?gene=ATP6V1G1) | ENSG00000136888.6 | 2.66E-10 |
| [RP11-89H19.2](http://gepia.cancer-pku.cn/detail.php?gene=RP11-89H19.2) | ENSG00000278385.1 | 2.71E-10 |
| [PIGV](http://gepia.cancer-pku.cn/detail.php?gene=PIGV) | ENSG00000060642.10 | 2.81E-10 |
| [PAQR5](http://gepia.cancer-pku.cn/detail.php?gene=PAQR5) | ENSG00000137819.13 | 2.97E-10 |
| [EPB41L4A-AS2](http://gepia.cancer-pku.cn/detail.php?gene=EPB41L4A-AS2) | ENSG00000278921.2 | 3.19E-10 |
| [DMXL1](http://gepia.cancer-pku.cn/detail.php?gene=DMXL1) | ENSG00000172869.14 | 3.20E-10 |
| [AQP1](http://gepia.cancer-pku.cn/detail.php?gene=AQP1) | ENSG00000240583.10 | 3.71E-10 |
| [AKTIP](http://gepia.cancer-pku.cn/detail.php?gene=AKTIP) | ENSG00000166971.16 | 3.85E-10 |
| [RP4-635A23.6](http://gepia.cancer-pku.cn/detail.php?gene=RP4-635A23.6) | ENSG00000279838.1 | 3.86E-10 |
| [AFG3L2](http://gepia.cancer-pku.cn/detail.php?gene=AFG3L2) | ENSG00000141385.9 | 3.90E-10 |
| [C9orf156](http://gepia.cancer-pku.cn/detail.php?gene=C9orf156) | ENSG00000136932.13 | 3.90E-10 |
| [FNIP2](http://gepia.cancer-pku.cn/detail.php?gene=FNIP2) | ENSG00000052795.12 | 4.09E-10 |
| [IQGAP2](http://gepia.cancer-pku.cn/detail.php?gene=IQGAP2) | ENSG00000145703.15 | 4.34E-10 |
| [SAA2](http://gepia.cancer-pku.cn/detail.php?gene=SAA2) | ENSG00000134339.8 | 4.46E-10 |
| [NBR1](http://gepia.cancer-pku.cn/detail.php?gene=NBR1) | ENSG00000188554.13 | 4.62E-10 |
| [PLEKHF2](http://gepia.cancer-pku.cn/detail.php?gene=PLEKHF2) | ENSG00000175895.3 | 4.69E-10 |
| [AUH](http://gepia.cancer-pku.cn/detail.php?gene=AUH) | ENSG00000148090.11 | 4.85E-10 |
| [TAL2](http://gepia.cancer-pku.cn/detail.php?gene=TAL2) | ENSG00000186051.6 | 4.87E-10 |
| [SLC2A9](http://gepia.cancer-pku.cn/detail.php?gene=SLC2A9) | ENSG00000109667.11 | 5.27E-10 |
| [OTX1](http://gepia.cancer-pku.cn/detail.php?gene=OTX1) | ENSG00000115507.9 | 6.24E-10 |
| [RSG1](http://gepia.cancer-pku.cn/detail.php?gene=RSG1) | ENSG00000132881.11 | 6.30E-10 |
| [ESD](http://gepia.cancer-pku.cn/detail.php?gene=ESD) | ENSG00000139684.13 | 6.37E-10 |
| [ILVBL](http://gepia.cancer-pku.cn/detail.php?gene=ILVBL) | ENSG00000105135.15 | 6.41E-10 |
| [SGCB](http://gepia.cancer-pku.cn/detail.php?gene=SGCB) | ENSG00000163069.12 | 6.49E-10 |
| [DLX4](http://gepia.cancer-pku.cn/detail.php?gene=DLX4) | ENSG00000108813.10 | 6.56E-10 |
| [RP11-2E11.9](http://gepia.cancer-pku.cn/detail.php?gene=RP11-2E11.9) | ENSG00000270953.1 | 6.58E-10 |
| [MYO6](http://gepia.cancer-pku.cn/detail.php?gene=MYO6) | ENSG00000196586.13 | 6.85E-10 |
| [FREM2](http://gepia.cancer-pku.cn/detail.php?gene=FREM2) | ENSG00000150893.10 | 7.25E-10 |
| [DMRT3](http://gepia.cancer-pku.cn/detail.php?gene=DMRT3) | ENSG00000064218.4 | 7.46E-10 |
| [RP11-16E12.2](http://gepia.cancer-pku.cn/detail.php?gene=RP11-16E12.2) | ENSG00000259772.6 | 7.71E-10 |
| [DENND1C](http://gepia.cancer-pku.cn/detail.php?gene=DENND1C) | ENSG00000205744.9 | 8.10E-10 |
| [IGBP1](http://gepia.cancer-pku.cn/detail.php?gene=IGBP1) | ENSG00000089289.15 | 8.19E-10 |
| [CCDC121](http://gepia.cancer-pku.cn/detail.php?gene=CCDC121) | ENSG00000176714.9 | 8.73E-10 |
| [MRPS18B](http://gepia.cancer-pku.cn/detail.php?gene=MRPS18B) | ENSG00000204568.11 | 8.84E-10 |
| [EZR](http://gepia.cancer-pku.cn/detail.php?gene=EZR) | ENSG00000092820.17 | 9.13E-10 |
| [DCST2](http://gepia.cancer-pku.cn/detail.php?gene=DCST2) | ENSG00000163354.14 | 9.35E-10 |
| [F2RL1](http://gepia.cancer-pku.cn/detail.php?gene=F2RL1) | ENSG00000164251.4 | 9.93E-10 |
| [MRPL50](http://gepia.cancer-pku.cn/detail.php?gene=MRPL50) | ENSG00000136897.7 | 1.06E-09 |
| [FAHD1](http://gepia.cancer-pku.cn/detail.php?gene=FAHD1) | ENSG00000180185.11 | 1.08E-09 |
| [KIF13B](http://gepia.cancer-pku.cn/detail.php?gene=KIF13B) | ENSG00000197892.12 | 1.10E-09 |

Table S2. Clinical characteristics of ccRCC patients with low/high expression of *PEBP1P2.*

| Characteristic | Low expression of PEBP1P2 (n=269) | High expression of PEBP1P2 (n=270) | *P* |
| --- | --- | --- | --- |
| Age | 61 (53 - 69) | 60 (51 - 70.75) | 0.526 |
| T stage |  |  | **0.003** |
| T1 | 118 (21.9%) | 160 (29.7%) |  |
| T2 | 41 (7.6%) | 30 (5.6%) |  |
| T3 | 102 (18.9%) | 77 (14.3%) |  |
| T4 | 8 (1.5%) | 3 (0.6%) |  |
| Pathologic stage |  |  | **0.003** |
| Stage I | 114 (21.3%) | 158 (29.5%) |  |
| Stage II | 32 (6%) | 27 (5%) |  |
| Stage III | 73 (13.6%) | 50 (9.3%) |  |
| Stage IV | 48 (9%) | 34 (6.3%) |  |
| Histologic grade |  |  | **0.002** |
| G1 | 3 (0.6%) | 11 (2.1%) |  |
| G2 | 103 (19.4%) | 132 (24.9%) |  |
| G3 | 109 (20.5%) | 98 (18.5%) |  |
| G4 | 48 (9%) | 27 (5.1%) |  |

Continuous variables are presented as median (interquartile range, IQR), while categorical variables are presented as patients (%). Significant *P* values were presented in bold text.

Table S3. Primers used for real-time PCR.

| Target  gene | Primer sequence (5’-3’) | | Size  (bp) |
| --- | --- | --- | --- |
|  | Forward | Reverse |  |
| *PEBP1P2* | ACTTTAGTGGCCTGTCCTGCTCA | TGACGCCCACCCAGGTTAAGAATA | 276 |
| *PEBP1* | TCTGGCTGGTTTACGAGCAG | CACCTTGAATTTGCCACGGT | 92 |
| *KLF13* | CGGCCTCAGACAAAGGGTC | TTCCCGTAAACTTTCTCGCAG | 105 |
| *18s rRNA* | CAGCCACCCGAGATTGAGCA | TAGTAGCGACGGGCGGTGTG | 252 |
| *GAPDH* | AACGGATTTGGTCGTATTGGG | CCTGGAAGATGGTGATGGGAT | 211 |
| *AR* | GACGACCAGATGGCTGTCATT | GGGCGAAGTAGAGCATCCT | 106 |
| *CEBPA* | GCGGCGACTTTGACTACCC | GCTGCTTGGCTTCATCCTCC | 210 |
| *CEBPB* | CTTCAGCCCGTACCTGGAG | GGAGAGGAAGTCGTGGTGC | 136 |
| *FOXP3* | GTGGCCCGGATGTGAGAAG | GGAGCCCTTGTCGGATGATG | 238 |
| *HOXD10* | AGACAGTTGGACAGATCCGAA | CGAAATGAGTTTGTTGCGCTTAT | 142 |
| *IRF1* | ATGCCCATCACTCGGATGC | CCCTGCTTTGTATCGGCCTG | 204 |
| *NR3C1* | ATAGCTCTGTTCCAGACTCAACT | TCCTGAAACCTGGTATTGCCT | 111 |
| *STAT4* | TGTTGGCCCAATGGATTGAAA | GGAAACACGACCTAACTGTTCAT | 119 |
| *TFAP2A* | GACTCGGAGACCTCTCGATCC | GACGGCATTGCTGTTGGAC | 143 |
| *TP53* | GAGGTTGGCTCTGACTGTACC | TCCGTCCCAGTAGATTACCAC | 133 |
| *EIF4A3* | AAGGGAGAGATGTCATCGCAC | GCTTGAGTTTCACGAACCTGA | 106 |
| *DHX9* | CGAACCATCTCAGCGACAAAA | TGAGGTCCATGCTTATTTGCTC | 140 |
| *FAM120A* | GAGGGGCGACAAACCATATCA | GCCTTCGGCTAGTTGGCTT | 149 |
| *IGF2BP2* | AGCTAAGCGGGCATCAGTTTG | CCGCAGCGGGAAATCAATCT | 176 |
| *FBL* | GTCTTCATTTGTCGAGGAAAGGA | AAATCGAGACTCTCTTCTCTCCA | 91 |
| *RBM47* | TGATGGACTTTGACGGCAAGA | GGGCGGATCTCGTAGTTGTT | 106 |
| *SRSF1* | GGAAGACGCGGTGTATGGTC | CACCTGCTTCACGCATGTG | 236 |
| *UPF1* | ACCGACTTTACTCTTCCTAGCC | AGGTCCTTCGTGTAATAGGTGTC | 248 |
| *ELAVL1* | AACTACGTGACCGCGAAGG | CGCCCAAACCGAGAGAACA | 194 |
| *YBX1* | CCCCAGGAAGTACCTTCGC | AGCGTCTATAATGGTTACGGTCT | 158 |

Table S4. Primers used for ChIP assay.

| Target  promoter | Primer sequence (5’-3’) | | Size  (bp) |
| --- | --- | --- | --- |
|  | Forward | Reverse |  |
| *PEBP1P2* site1 | TGAAGCGGAACTTGTACTCAG | TTCAGGCATCAGAACCAA | 198 |
| *PEBP1P2* site2 | AATCAAGCCTTATCCACCA | GACTTCCCTCCCTTACCC | 179 |
| *PEBP1P2* site3 | AGGTGAATGCAAGCTCAA | TGCAAATGCCATCACAAT | 193 |

Table S5. Primers used for RNA modification RIP-qRT-PCR analysis.

| Target  transcript | Primer sequence (5’-3’) | | Size  (bp) |
| --- | --- | --- | --- |
|  | Forward | Reverse |  |
| *PEBP1* ac^4^C site1 primer1 | TCTCCGCGTCGCCTCTGT | TTGCCCAGCTCGTCCACC | 190 |
| *PEBP1* ac^4^C site1 primer2 | TCTCCGCGTCGCCTCTGT | ACCTGGGTGGGCGTCAGCA | 211 |
| *PEBP1* ac^4^C site2 primer1 | GTTGCCTCTGCCTTTGTG | GATCTCAGGTTTCCTCCC | 272 |
| *PEBP1* ac^4^C site2 primer2 | AGATTGGTTGCCTCTGCC | GATCTCAGGTTTCCTCCC | 278 |
| *PEBP1* m^6^A site1 primer1 | CCGGTGGACCTCAGCAAGT | GGTGGGCGTCAGCACTTT | 129 |
| *PEBP1* m^6^A site1 primer2 | CCTTGAGCCTGCAAGAAG | TAACCTGGGTGGGCGTCAGC | 108 |
| *PEBP1* m^6^A site2 primer1 | AGACCACCGTGGCAAATT | GGCCAGGCAGCAGGAAAA | 298 |
| *PEBP1* m^6^A site2 primer2 | AGACCACCGTGGCAAATT | AAGGCCAGGCAGCAGGAA | 300 |
| *PEBP1* m^6^A site3 primer1 | AGTCAGATGGTAGTTGAGGGTG | GATCACAAAGGCAGAGGC | 292 |
| *PEBP1* m^6^A site3 primer2 | AAATAGCAACCCAGAATGTA | GCCTCAATGCCAGCCTCT | 221 |
| *PEBP1* m^5^C site1 primer1 | TCTCCGCGTCGCCTCTGT | TTGCCCAGCTCGTCCACC | 190 |
| *PEBP1* m^5^C site1 primer2 | TCTCCGCGTCGCCTCTGT | ACCTGGGTGGGCGTCAGCA | 211 |
| *PEBP1* m^5^C site2 primer1 | GTTGCCTCTGCCTTTGTG | GATCTCAGGTTTCCTCCC | 272 |
| *PEBP1* m^5^C site2 primer2 | AGATTGGTTGCCTCTGCC | GATCTCAGGTTTCCTCCC | 278 |

Table S6. Probes used for RNA FISH and RNA pulldown.

| Target transcript | Probe sequence (5’-3’) |
| --- | --- |
| *PEBP1P2* FISH probe | CCTGCTGGGAGCATCAGGGTCTGTCAGGACCAA |
| *PEBP1P2* RNA pulldown probe | GGAAATGATGCCATTCTCTGTATTTGGGATCCTTC |
| *PEBP1* RNA pulldown probe | GGTACTGTGATGGGGTCATCAAATTATTAATCTGA |
| *PEBP1* RNA pulldown probe | ACTCACTCACTCTGATTTATGTTTTGATCAAATTT |

Table S7. SiRNA, shRNA and ASOs used for silencing target genes.

| Target transcript | Sequence (5’-3’) | |
| --- | --- | --- |
| *PEBP1P2* sh1#  *PEBP1P2* sh2#  *PEBP1* sh1#  *PEBP1* sh2#  *KLF13* sh1#  *KLF13* sh2#  *AR* sh1#  *AR* sh2#  *CEBPA* sh1#  *CEBPA* sh2#  *CEBPB* sh1#  *CEBPB* sh2#  *FOXP3* sh1#  *FOXP3* sh2#  *HOXD10* sh1#  *HOXD10* sh2#  *IRF1* sh1#  *IRF1* sh2#  *NR3C1* sh1#  *NR3C1* sh2#  *STAT4* sh1#  *STAT4* sh2#  *TFAP2A* sh1#  *TFAP2A* sh2#  *TP53* sh1#  *TP53* sh2#  *EIF4A3* sh1#  *EIF4A3* sh2#  *DHX9* sh1#  *DHX9* sh2#  *FAM120A* sh1#  *FAM120A* sh2#  *IGF2BP2* sh1#  *IGF2BP2* sh2#  *FBL* sh1#  *FBL* sh2#  *RBM47* sh1#  *RBM47* sh2#  *SRSF1* sh1#  *SRSF1* sh2#  *UPF1* sh1#  *UPF1* sh2#  *ELAVL1* sh1#  *ELAVL1* sh2#  *YBX1* sh1#  *YBX1* sh2# | GCTCATAAACCAGCCAGACTT  CCATTTCTCGCAGGCTCAATT  CGAGCAGCTGTCTGGGAAGTA  GTGGTCAACATGAAGGGCAAT  GCGAGAAAGTTTACGGGAAAT  GCGAGAAAGTTTACGGGAAAT  CACCAATGTCAACTCCAGGAT  CACCAATGTCAACTCCAGGAT  GCTGGAGCTGACCAGTGACAA  CAAGAAGTCGGTGGACAAGAA  CCCGTGGTGTTATTTAAAGAA  CCTGCCTTTAAATCCATGGAA  CACACGCATGTTTGCCTTCTT  CCTCCACAACATGGACTACTT  GAGATCAGTAAGAGCGTTAAC  TCGTAATGCAGGGTAACTATT  CCTCTGTCTATGGAGACTTTA  GCGTGTCTTCACAGATCTGAA  GTGTCACTGTTGGAGGTTATT  CACAGGCTTCAGGTATCTTAT  CCTGCCACATTGAGTCAACTA  GCGAGACTACAAAGTTATTAT  CCAATGAGCAAGTGACAAGAA  CCCAGATCAAACTGTAATTAA  CGGCGCACAGAGGAAGAGAAT  GTCCAGATGAAGCTCCCAGAA  GCTCTCGGTGACTACATGAAT  GCAATCAAGCAGATCATCAAA  ACGACAATGGAAGCGGATATA  TTAAGGAAACCAAGCATATAG  GCCTTGAATAATGACTCTAAA  GCTTCCTTTCACTGGAGTTTA  AGTGAAGCTGGAAGCGCATAT  GGTGCCTGCAGCGGTAATATA  GCAGCAGACGTGCTACAATTT  CGGCAGAAAGTTTGGAAGCAA  GATGAAGAAGCGCGAGGAAAT  CGCTTGACTATTTATGAAGAA  GAAGCAGGTGATGTATGTTAT  TAAAGCTCCTGTTACTCATAT  GCCTACCAGTACCAGAACATA  CCAACCCGATAAACCGATGTT  TACCAGTTTCAATGGTCATAA  CGAGCTCAGAGGTGATCAAAG  CCAGTTCAAGGCAGTAAATAT  AGCAGACCGTAACCATTATAG |  |

Table S8. Guide RNA used for SAM system and targeted RNA methylation system.

| Target promoter | gRNA sequence (5’-3’) |
| --- | --- |
| *PEBP1P2* gRNA1 | AAACAAAACTATTATTGTGA |
| *PEBP1P2* gRNA2 | AATTTCAAATGTTAAAGCAA |
| *PEBP1P2* gRNA3 | AATTATAAAGTTACAAAAAG |
| Target transcript | gRNA sequence (5’-3’) |
| *PEBP1* gRNA1 | TGAGTAAAATTATAAAGGCCAGG |
| *PEBP1* gRNA2 | GAGTAAAATTATAAAGGCCAGGC |
| *PEBP1* gRNA3 | AAAATTATAAAGGCCAGGCAGCA |

Table S9. Primary antibodies used in this study.

| Source | Primary antibodies | Catalog no. | Working dilution |
| --- | --- | --- | --- |
| ProteinTech | Anti-STAT4 antibody produced in rabbit | 13028-1-AP | WB: 1:1000 IHC:1:200  ChIP: 5μg |
| ProteinTech | Anti-KLF13 antibody produced in rabbit | 18352-1-AP | WB: 1:1000 IHC:1:200 |
| ProteinTech | Anti-YBX1 antibody produced in rabbit | 20339-1-AP | WB: 1:1000 IHC:1:200  RIP: 5μg |
| ProteinTech | Anti-ELAVL1 antibody produced in rabbit | 11910-1-AP | WB: 1:1000 IHC:1:200  RIP: 5μg |
| ProteinTech | Anti-PEBP1 antibody produced in rabbit | 10575-1-AP | WB: 1:1000 IHC:1:200 |
| ProteinTech | Anti-EIF4A3 antibody produced in rabbit | 17504-1-AP | WB: 1:1000 RIP: 5μg |
| ProteinTech | Anti-DHX9 antibody produced in rabbit | 17721-1-AP | WB: 1:1000 RIP: 5μg |
| ProteinTech | Anti-FAM120A antibody produced in rabbit | 21529-1-AP | WB: 1:1000 RIP: 5μg |
| ProteinTech | Anti-IGF2BP2 antibody produced in rabbit | 11601-1-AP | WB: 1:1000 RIP: 5μg |
| ProteinTech | Anti-FBL antibody produced in rabbit | 16021-1-AP | WB: 1:1000 RIP: 5μg |
| ProteinTech | Anti-RBM47 antibody produced in rabbit | 23902-1-AP | WB: 1:1000 RIP: 5μg |
| ProteinTech | Anti-SRSF1 antibody produced in rabbit | 12929-2-AP | WB: 1:1000 RIP: 5μg |
| ProteinTech | Anti-UPF1 antibody produced in rabbit | 23379-1-AP | WB: 1:1000 RIP: 5μg |
| ProteinTech | Anti-GFP antibody produced in rabbit | 50430-2-AP | WB: 1:2500 RIP: 5μg |
| ABclonal | Anti-ACTB antibody produced in rabbit | AC026 | WB: 1:15000 |
| Abcam | Anti-m^6^A antibody produced in mouse | ab151230 | RIP: 5μg |
| Diagenode | Anti-m^5^C antibody produced in mouse | C15200003 | RIP: 5μg |
| Abcam | Anti-ac^4^C antibody produced in mouse | ab252215 | RIP: 5μg |
| Cell Signaling Technology | Anti-AGO2 antibody produced in rabbit | 2897 | RIP: 5μg |

Table S10. Small-molecule inhibitors used in this study.

| Source | Small-molecule inhibitors | Catalog no. | Working dilution |
| --- | --- | --- | --- |
| MedChemExpress | Cycloheximide | HY-12320 | 5 µM |
| MedChemExpress | α-Amanitin | HY-19610 | 50 µg/mL |
